# From root to embryogenic transition: WOX5 reprograms plant somatic cells via auxin-mediated pathways

**DOI:** 10.1186/s12870-025-06687-4

**Published:** 2025-05-15

**Authors:** Anna M. Wójcik, Kamila Krypczyk, Weronika M. Buchcik, Małgorzata D. Gaj

**Affiliations:** University of Silesia in Katowice, Faculty of Natural Sciences, Institute of Biology, Biotechnology and Environmental Protection, Jagiellonska 28, Katowice, 40-032 Poland

**Keywords:** Somatic embryogenesis, Auxin, WOX5, WUSCHEL-RELATED HOMEOBOX, Stem cells, Pluripotency, TAA1, CDF4, PLETHORA, PLT, PIN, ARF5, 2,4-D, Embryogenesis, Arabidopsis

## Abstract

The study was focused on the WUSCHEL-RELATED HOMEOBOX 5 (WOX5), a member of the WUSCHEL family of homeodomain transcription factors, of the root apical meristem (RAM) – specific function in the quiescent center (QC) and columella stem cell maintenance. We revealed that WOX5 is also engaged in the embryogenic transition of plant somatic cells cultured in vitro. We showed that WOX5 controls the induction of somatic embryogenesis (SE) in Arabidopsis. The results suggest that the function of WOX5 in SE induction is related to controlling genes related to diverse auxin-related processes, including biosynthesis, transport, distribution, and signaling of auxin in plant development (*TAA1, YUC1, PIN1, LEC2, PLT3, ARF5*). The postulated WOX5 targets in embryogenic induction also involve *CDF4,* controlling cell differentiation in RAM. The study reveals genetic parallels between stem cell maintenance in RAM and in vitro-induced embryogenic transition in somatic cells.

The findings identified WOX5 as a new regulatory element within the transcription factor network controlling embryogenic response in somatic plant cells. Identifying embryogenic/pluripotency-related functions of WOX5 opens further opportunities for improving in vitro plant regeneration of recalcitrant species.

**Supplementary Information:**

The online version contains supplementary material available at 10.1186/s12870-025-06687-4.

## Introduction

Plants exhibit extraordinary developmental plasticity, allowing them, by tissue and organ regeneration, to cope with external cues and environmental challenges [1]. This unique plant ability has been extensively studied and exploited in modern biotechnology, facilitating both clonal propagation and genetic modification of plants for plant breeding and functional genomics [2, 3]. Somatic embryogenesis (SE) and de novo shoot organogenesis are the major morphogenic processes through which plants regenerate from in vitro cultured somatic cells [4]. The SE induction mechanism attracts research attention to understanding the molecular mechanisms governing plant totipotency. Studies on embryogenic transition in the SE-induction system established for a model plant of *Arabidopsis thaliana* remain a main focus in research on plant developmental plasticity and cell totipotency [5, 6].

SE is a complex, multi-step, and plant-specific developmental process. It involves reprogramming somatic cells towards the acquisition of embryogenic competence and triggering an embryogenic program, resulting in the formation of somatic embryos [7, 8]. The embryogenic transition of somatic cells is initiated by external stimuli such as plant growth regulators (PGRs), particularly auxins and cytokinins, which re-direct a cell fate [9, 10]. Advanced studies on the genetic mechanism of SE induction in Arabidopsis revealed that the somatic-to-embryogenic transition of explant cells is associated with extensive reprogramming of the somatic cell transcriptome [11]. The core regulatory network orchestrating the embryogenic transition at the transcriptomic level involves transcription factor (TF) genes that cooperate with diverse epigenetic processes, including chromatin remodeling and miRNA-dependent regulation [12–14]. The main processes controlled by SE-engaged TFs were attributed to the regulation of cell proliferation, zygotic embryogenesis, and stem cell maintenance. The SE-engaged TF targets were found to be mainly involved in auxin- and stress-related responses [15]. The core elements of the TF-network controlling SE induction include LEAFY COTYLEDON1 (LEC1), LEC2, FUSCA3 (FUS3), MYB118, SHOOT MERISTEMLESS1 (STM1); AGAMOUS-LIKE15 (AGL15), PLETHORA4/BABY BOOM (PLT4/BBM), PLT5/EMBRYO MAKER (PLT5/EMK), WUSCHEL (WUS), and WUSCHEL-related homeobox (WOX) TFs [14, 16].

The *WUS/WOX* genes form a plant-specific subclade of the eukaryotic homeobox TF superfamily, characterized by a conserved DNA-binding homeodomain (HD). Besides *WUS*, fourteen *WOX* (*WOX1-14*) genes, grouped into three phylogenetic clades, were identified in Arabidopsis and rice genomes [17]. The prototypic WUS and other WOX members of the WUS/WOX TF family regulate plant development by controlling stem cell maintenance, embryonic patterning, and organ formation by the promotion of cell division activity and/or the prevention of premature cell differentiation [18, 19]. Two of the WUS/WOX proteins, the WUSCHEL (WUS) and WUSCHEL-RELATED HOMEOBOX 5 (WOX5) have master regulatory roles in plant development as they induce and maintain the central stem cell populations within the shoot apical meristem (SAM) and root apical meristem (RAM), respectively [20]. WUS and WOX5 show intercellular mobility, enabling them to regulate stem cell niches in apical meristems non-cell-autonomously [21]. Despite their primary roles in distinct meristems, WOX5 and WUS were shown to be exchangeable in regulating stem cell maintenance in the shoot and root [20]. Overexpression of the *LhWOX5*, an ortholog of *AtWOX5* in *Liriodendron*, resulted in ectopic flower formation, providing insight into the potential role of RAM-specific WOX5 in the development of the shoot [22]. The conserved WUS/WOX5 functions in angiosperm shoot/root stem-cell maintenance and floral organ formation were postulated [18, 21].

Consistent with controlling plant development in vivo, functions of *WUS/WOX* genes were also attributed to morphogenic processes induced in vitro in plant somatic cells. *WUS* activation in cultured explants of different plants promoted callus and somatic embryo development [23–25]. Similarly, the role of its close relative, WOX5, in callus induction and maintenance [26–28] and de novo shoot regeneration [29, 30] was reported. Moreover, *MtWOX9-1* overexpression improved SE efficiency in *Medicago truncatula* [31], and the combined ectopic expression of *WOX2* with either *WOX8* or *WOX9* in tobacco cell cultures enhanced plant regeneration processes [32]. In contrast, *WOX13* negatively regulated shoot regeneration from callus in *Arabidopsis thaliana* by inhibiting SAM formation [33].

These reports evidenced that different members of the *WUS/WOX* gene family control the induction of pluripotency in somatic cells cultured in vitro. However, how the *WOX* genes work and interplay with other regulatory SE-network components to promote embryogenic cell formation remains unknown.

The recent studies shed new light on the WOX5-related establishment of pluripotent stem cells in in vitro cultured explants. It was revealed that the quiescent center-related gene network (*SCR-SHR-WOX5-PLT1/2-JK*) with a central role of *WOX5* plays an essential role in callus induction in response to auxin treatment [27]. The WOX5-related gene network controls the division of callus founder cells to initiate the callus primordium and prevent callus tissue from differentiating into xylem cells in the culture of Arabidopsis hypocotyl explants treated with auxin [27]. This finding agrees with the observation that callus resembles a root meristem, even if it is derived from stem explants [26]. Besides auxin-promoted callus induction, WOX5 might contribute to the cytokinin-induced de novo shoot regeneration by repressing ARRs, negative regulators of cytokinin signaling [30]. Recently, a model of the cleavage of WOX5 protein by the peptidase DA1 was postulated to connect cytokinin signaling and root stem cell function [34].

Considering that the auxin-related TFs play crucial roles in the SE induction mechanism [9] and that WOX5 controls the stem cells and pluripotency by auxin-dependent mechanism [35], we found it interesting to get insight into the impact of this TF in the embryogenic transition induced in a model in vitro culture system for genetic studies on SE established in Arabidopsis [36]. Our results indicated that *WOX5* overexpression might recompense the auxin treatment required for SE induction. We revealed that WOX5 contributes to the embryogenic transition by impacting various auxin-related processes, including biosynthesis, transport, and auxin signaling. Within the postulated targets of WOX5 in SE, we indicated *LEC2*, *PLT3*, and *ARF5.* Moreover, the WOX5-mediated repression of cell differentiation factor CDF4 contributes to the SE induction mechanism. The results expand our knowledge of the TF-governed regulatory network controlling SE induction and indicate its new component, WOX5. Identifying embryogenic functions related to WOX5 opens further opportunities for improving SE induction and in vitro plant regeneration in different species.

## Results

### WOX5 positively controls the embryogenic potential of explants in an auxin-dependent manner

To investigate the role of *WOX5* in the auxin-induced SE process from Arabidopsis immature zygotic embryos (IZEs), we analyzed in vitro culture responses of the *35S::WOX5-GLUCOCORTICOID RECEPTOR* (*GR*) transgenic explants (hereafter called WOX5-GR). This transgenic system offers posttranscriptional control of TF function. Dexamethasone (DEX) treatment enables WOX5-GR fusion TF protein to enter the nucleus and transcriptionally regulate the WOX5 targets.

**Fig. 1:**
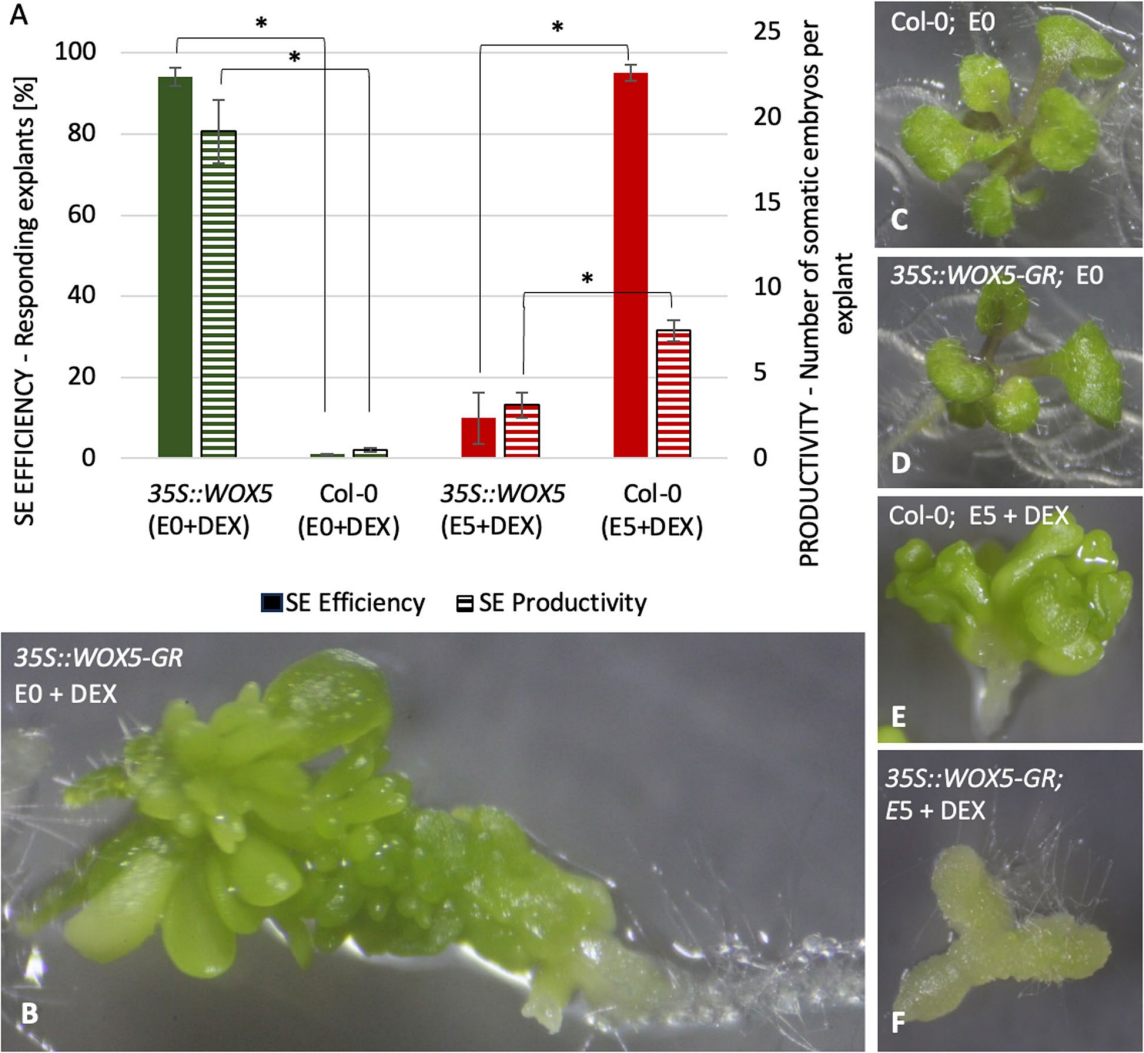
The embryogenic potential of the *35S::WOX5-GR* overexpression and Col-0 explants cultured on auxin-free E0 (green) and auxin E5 (red) medium with DEX (**A**). Asterisk (*)—values significantly differ from the Col-0 (WT) genotype (*P* < 0.05; *n* = 3 ± standard error). The phenotype of *35S::WOX5-GR* (**B**, **D**, **F**) and (**C**, **E**) explants after 21 days on E0 (**C**, **D**), E0 + DEX (**B**), E5 + DEX (**E**, **F**) medium. Solid bars: SE efficiency; Striped bars: SE productivity; d -day of SE culture

Given the essential role of auxin in SE induction, the responses of transgenic explants concerning auxin treatment were analyzed (Fig. 1A, B). In contrast to WT (Col-0), we found that the WOX5-GR explants treated with DEX underwent efficient SE induction on an auxin-free (E0) medium. Almost 90% of transgenic explants developed 20 somatic embryo-like structures per explant on average. The embryo-like structures transferred to ½ MS medium gave rise to fully developed seedlings with shoots and roots confirming their somatic embryo identity (Supp. Fig. S1). The embryogenic response of the WOX5-GR explants was inhibited on an auxin E5 medium where DEX-treated transgenic explants efficiently produced nonembryogenic callus (Fig. 1F).

**Fig. 2:**
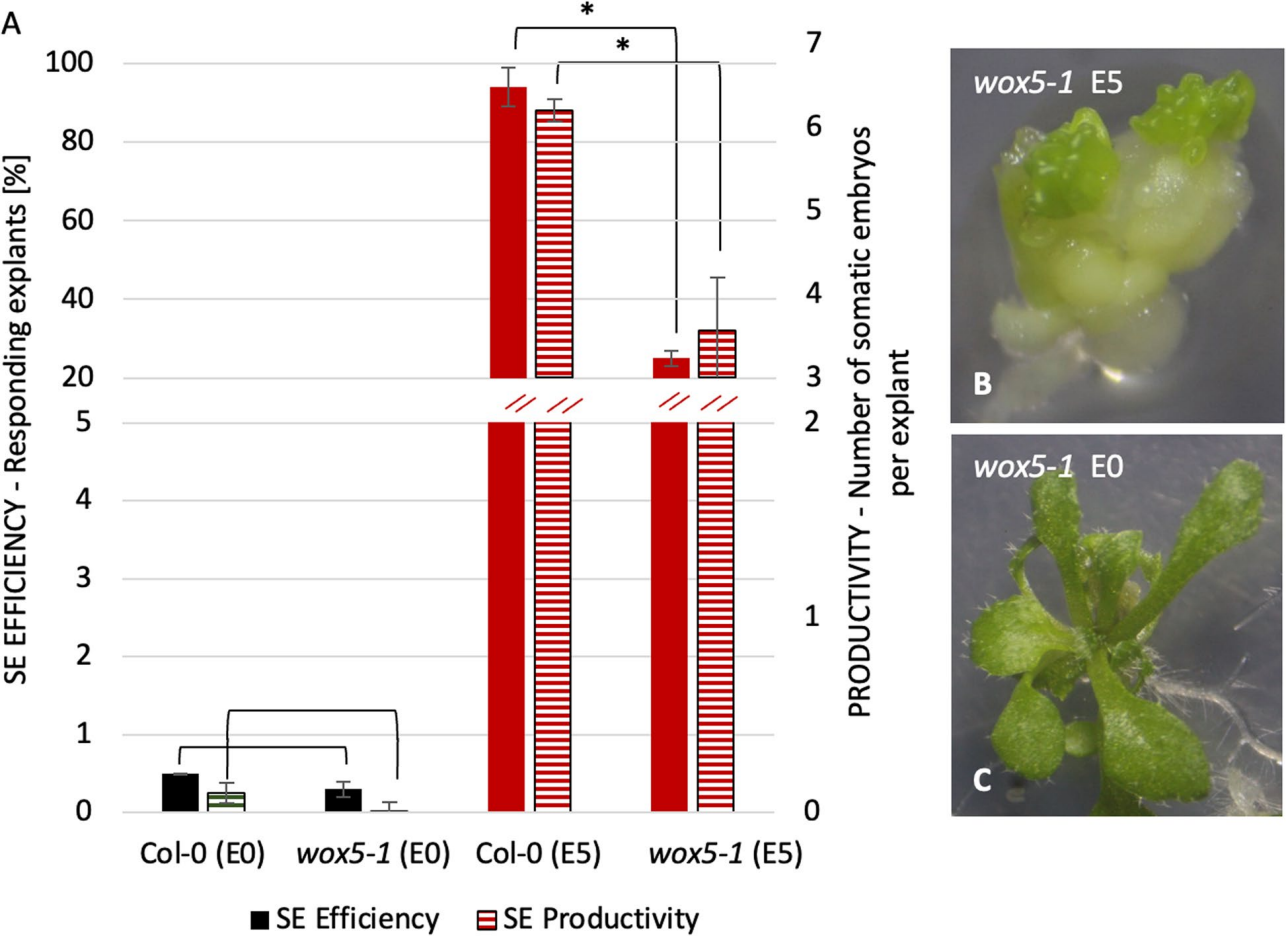
The embryogenic potential of the *wox5-1* insertional line and Col-0 (WT) explants cultured on auxin-free E0 (green) and auxin E5 (red) medium (**A**). Asterisks (*)—values significantly different from the Col-0 culture (*P* < 0.05; *n* = 3 ± standard error). The phenotype of *wox5-1* explants after 21 days on E5 (**B**, **D**, **F**) and E0 (**C**, **E**) medium. Solid bars: SE efficiency; Striped bars: SE productivity; d -day of SE culture

Further evidence of an engagement of *WOX5* in SE regulation provided the analysis of the embryogenic culture of the *wox5-1* line carrying insertion in the 3'UTR of *WOX5* (Fig. 2 A). We observed that *wox5-1* explants, similar to Col-0, were unable for SE induction on auxin-free E0 medium and developed into seedlings (Fig. 2 B). Auxin E5 medium that efficiently induced SE response in 98% of the Col-0 explants triggered an embryogenic response in only a tiny fraction (20%) of *wox5-*1 explants (Fig. 2 C).

**Fig. 3:**
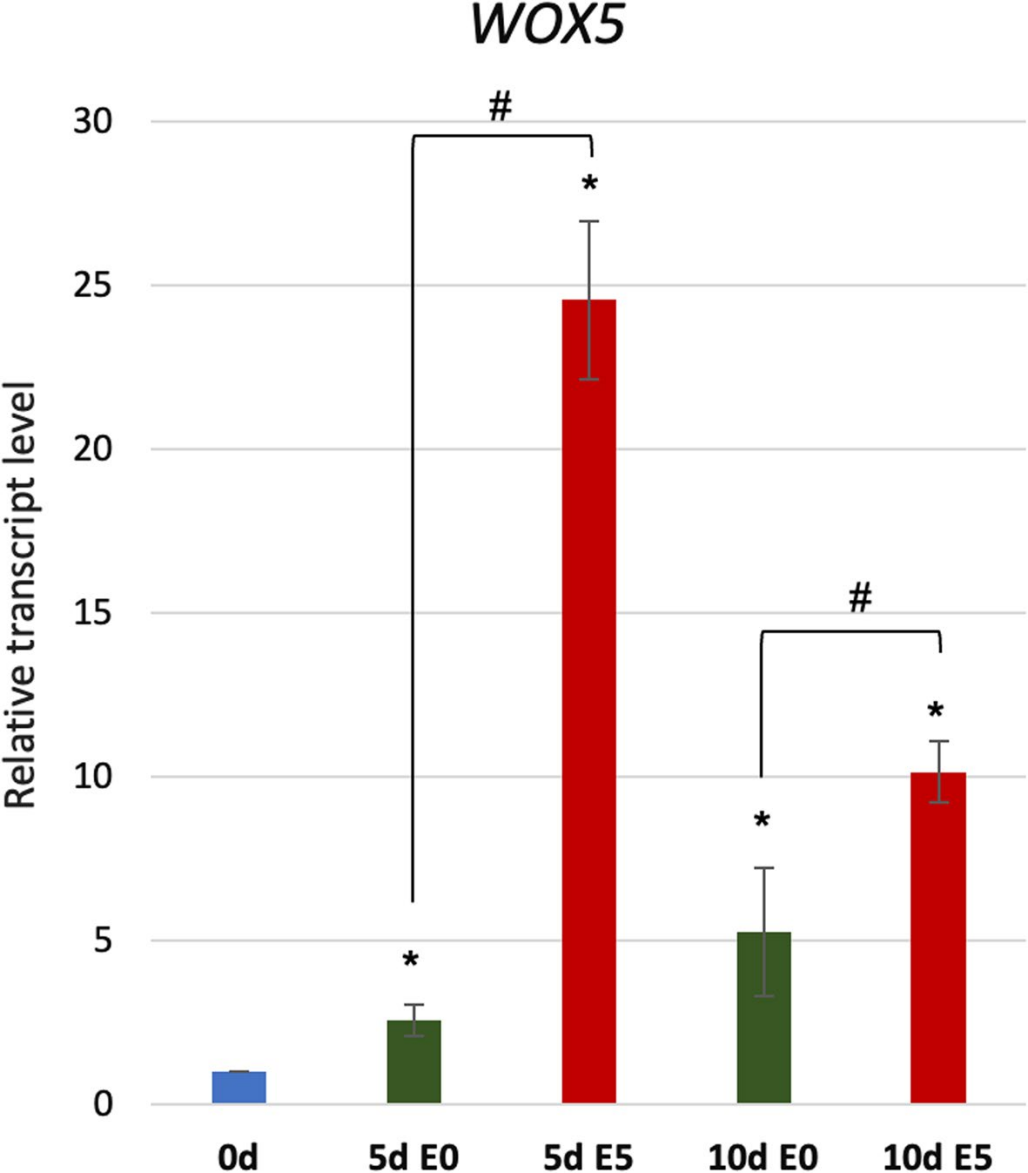
Expression level of *WOX5* in Col-0 (WT) explants cultured on auxin-free (E0) and auxin (E5) medium. The relative transcript level was normalized to the internal control (*At4g27090*) and calibrated to 0 days of the culture. Statistical analyses were performed using two-way ANOVA (*P* < 0.05) followed by Tukey's honestly significant difference test (Tukey HSD-test) (*P* < 0.05). Asterisk (*)—significantly different values to 0 d; hash (#)—significant differences between E5 and E0 culture of the same age (*P* < 0.05; *n* = 3 ± standard error); d -day of SE culture

Together, the results on explants with the disturbed WOX5 function (WOX5-GR and *wox5-1*) suggested a positive and auxin-related role of *WOX5* in SE induction. Supportive for this hypothesis were *WOX5* expression profiles in E0 and E5 Col-0 cultures. The results of real-time qRT-PCR analysis indicated a significant increase of *WOX5* expression in the embryogenic E5 culture (up to 24-fold). In contrast, the *WOX5* expression was almost 5 times lower on nonembryogenic E0 medium stimulating seedling development (Fig. 3).

**Fig. 4:**
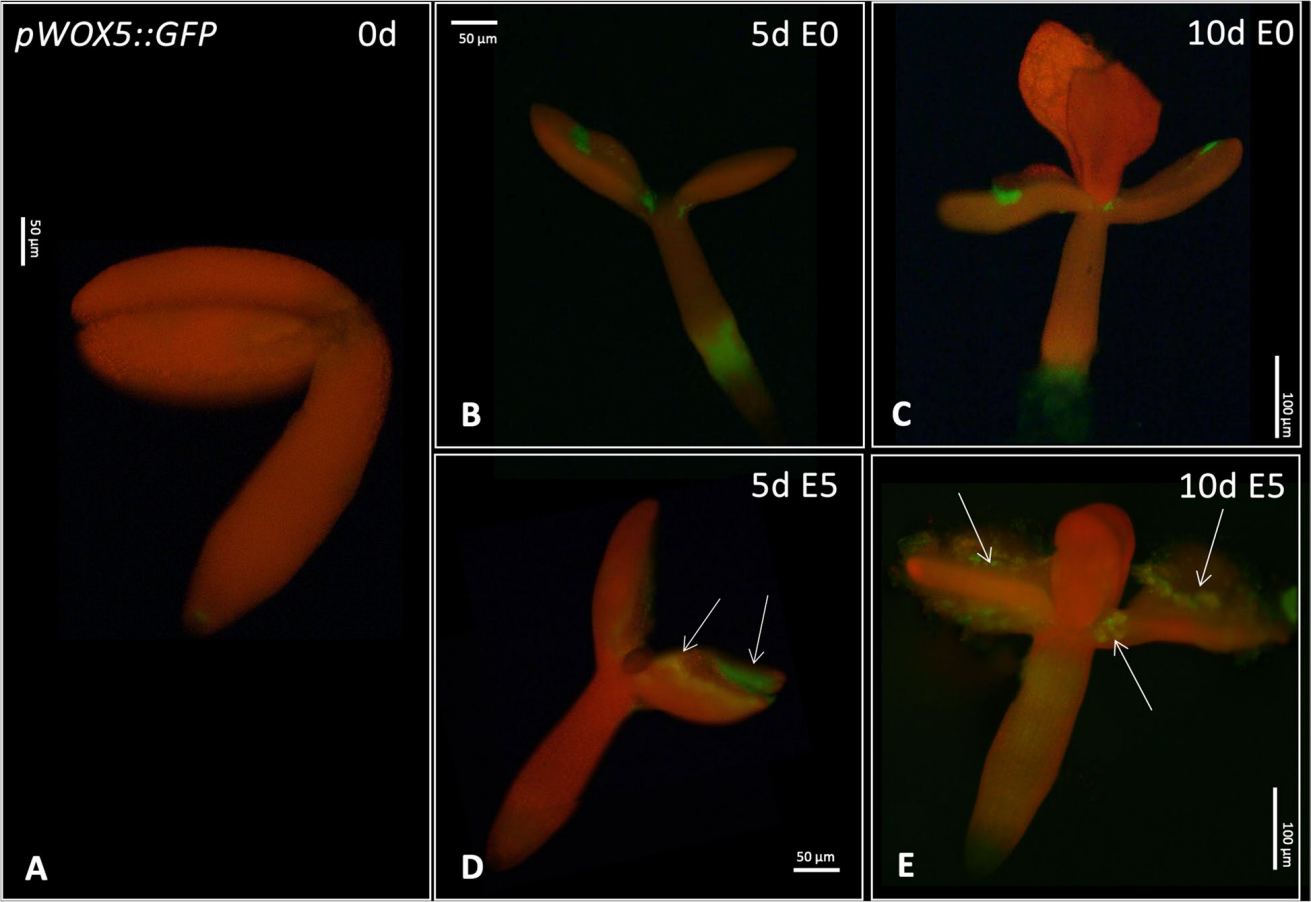
Spatio-temporal expression pattern of *WOX5* in explants cultured on auxin-free E0 (**B**, **C**) and auxin E5 (**D**, **E**) medium at 0, 5, and 10 d. Arrows indicate the GFP signal associated with the SE-involved tissue; d -day of SE culture

The spatiotemporal pattern of *WOX5* expression in the embryogenic vs. non-embryogenic culture was monitored using the *pWOX5::GFP* explants. The analysis showed that in freshly isolated explants (0 d), the GFP signal was limited to the RAM (Fig. 4 A) in agreement with the root-related *WOX5* function [37]. The culture of the explants on auxin E5 medium distinctly affected the *WOX5* expression pattern, and intensive GFP signals were also detected in the cotyledons of SE-induced explants (5 and 10 d culture). Less intensive GFP signals were noticed in hypocotyl (10 d) that are not involved in SE (Fig. 4. B-E). Colocalization of *WOX5* expression with cotyledon explant parts involved in SE-induction [38] further supported the SE-related functions of WOX5.

### WOX5 contributes to auxin biosynthesis and transport in SE

**Fig. 5:**
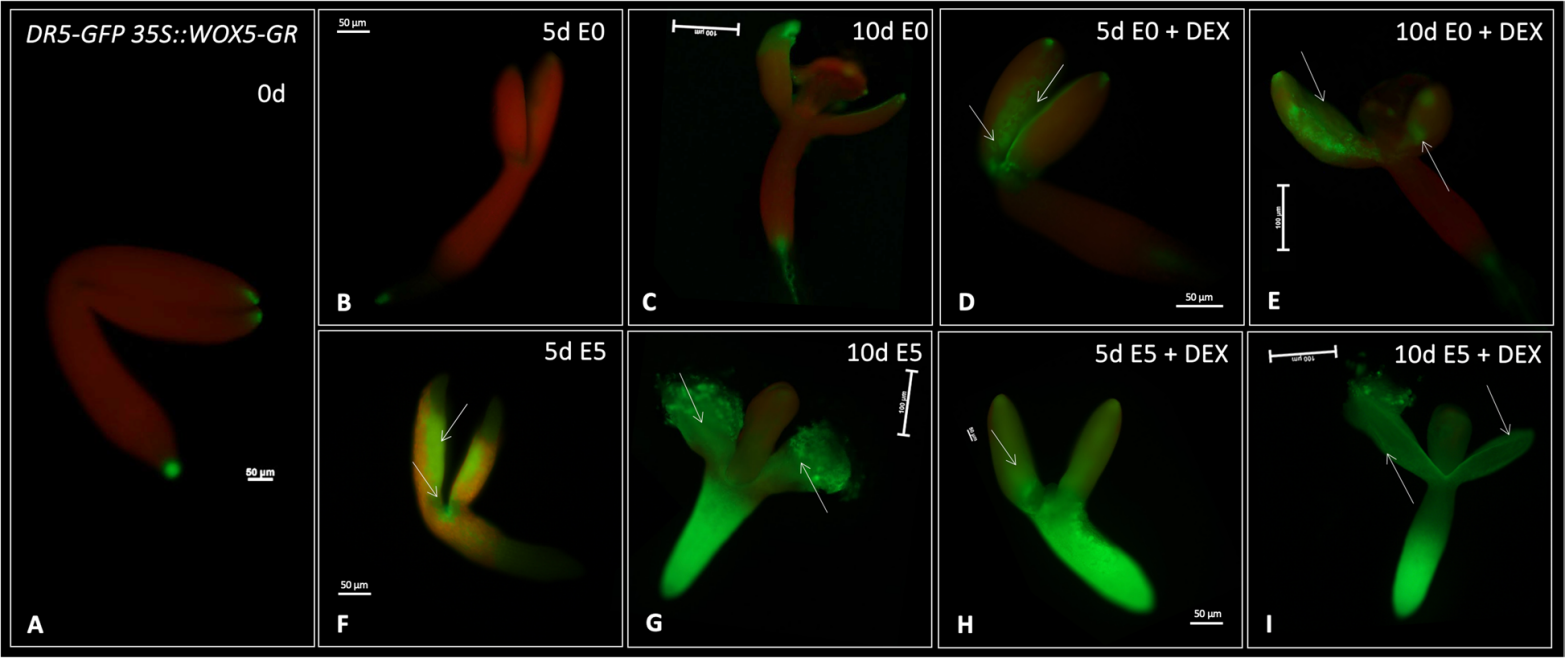
Spatio-temporal signal pattern of *DR5* in explants cultured on auxin-free E0 (**B**, **C**) and auxin E5 (**F**, **G**) medium supplemented (**D**, **E**), and not supplemented (H, I) with DEX at 0, 5, and 10-day culture (0, 5, 10 d). Arrows indicate the DR5-GFP signal associated with the SE-involved tissue

The auxin-related functions of WOX5 in SE were further investigated by monitoring auxin response under *WOX5* overexpression in the cultured explants (Fig. 5). We indicated an increase of DR5-controlled GFP signals in the explants overexpressing *WOX5* and cultured on auxin-free E0 medium. Notably, *WOX5* overexpression stimulated auxin response in SE-involved adaxial parts of explant cotyledons in E0 culture (Fig. 5D). Explant treatment with E5 resulted in intensive accumulation of DR5 signals, possibly due to the presence of auxin in the medium. Noteworthy, DR5 signals in early E5 culture (5 d) seemed much stronger under *WOX5* overexpression induced by DEX (Fig. 5 H, I) compared to the culture without DEX-induced *WOX5* overexpression (Fig. 5 F, G), which aligns with the assumed positive WOX5 impact on auxin accumulation in the cultured explants. The results confirmed a hypothesis on the auxin-related mechanism of WOX5 function in SE induction.

**Fig. 6:**
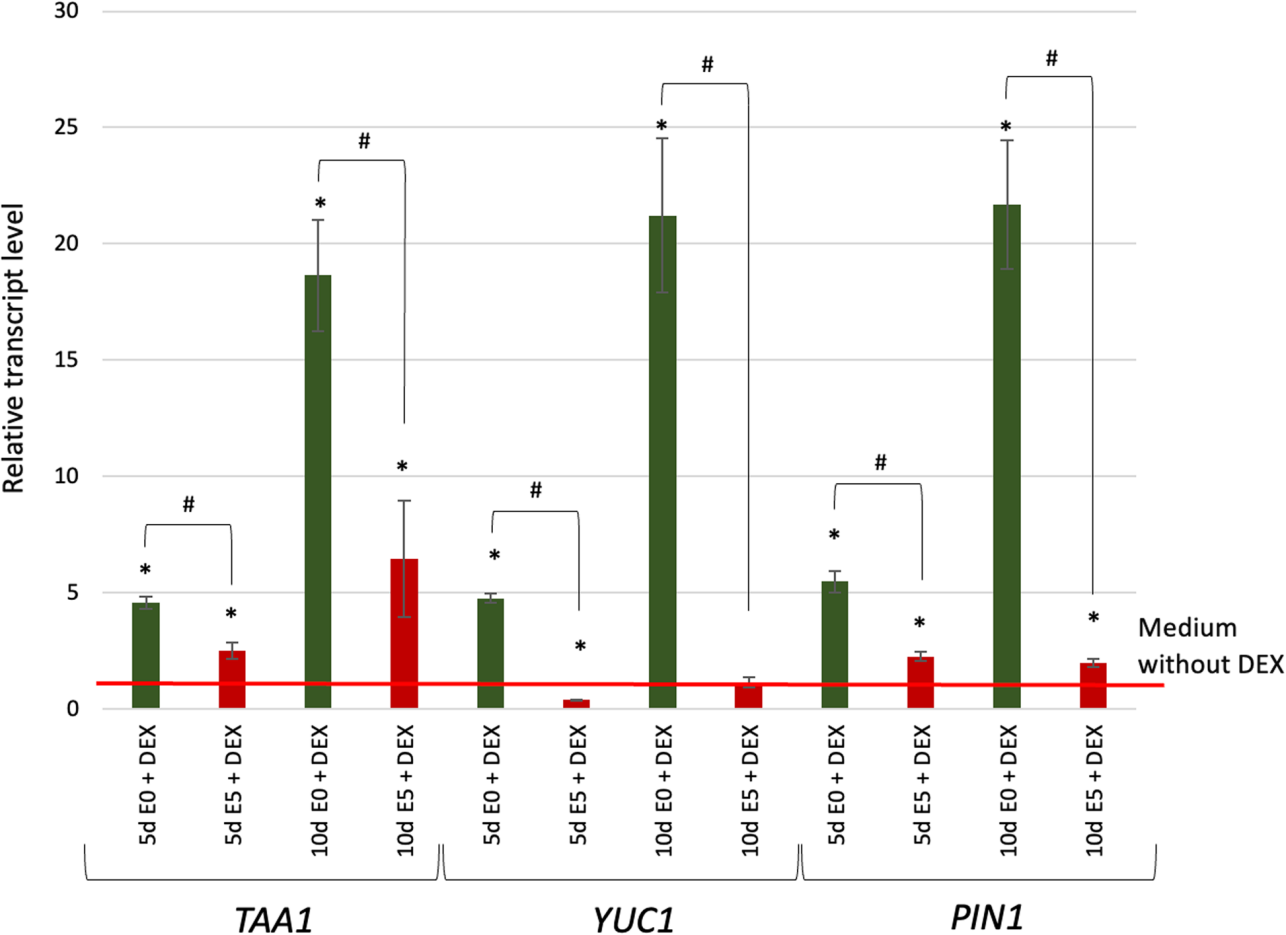
*WOX5* regulates the *TAA1, YUC1,* and *PIN1* gene expression during SE. The expression level of *TAA1, YUC1,* and *PIN1* in *35S::WOX5-GR* explants cultured on auxin-free (E0) and auxin (E5) medium supplemented with DEX (+ DEX) in relevance to DEX-free media. The relative transcript level was normalized to the internal control (*At4g27090*). Statistical analyses were performed using two-way ANOVA (*P* < 0.05) followed by Tukey's honestly significant difference test (Tukey HSD-test) (*P* < 0.05.). Asterisk (*)—significantly different values to DEX-free culture; hash (#)—significant differences between E5 + DEX and E0 + DEX culture of the same age (*P* < 0.05; *n* = 3 ± standard error); d -day of SE culture

WOX5 may promote auxin accumulation by controlling biosynthesis and/or transport of auxin. Thus, we evaluated the transcription level of *TRYPTOPHAN AMINOTRANSFERASE OF ARABIDOPSIS1* (*TAA1*) encoding a core enzyme, *YUCCA1* (*YUC1*) flavin monooxygenase in the tryptophan-dependent pathway of auxin biosynthesis, and *PIN-FORMED1* (*PIN*) auxin transporter in plant development and SE induction [39–41]. *TAA1, YUC1,* and *PIN1* expression levels in relevance to *WOX5* overexpression and auxin treatment were evaluated (Fig. 6). The results indicated that overexpression of *WOX5* resulted in a higher transcription level of the *TAA1* and *PIN1* genes in explants both treated (E5) and untreated (E0) with auxin, while the *YUC1* expression was increased only on E0 medium upon DEX induced *WOX5* overexpression. The results suggested the engagement of WOX5 in regulating the TAA1/YUC1-controlled auxin biosynthesis pathway and auxin transport.

**Fig. 7:**
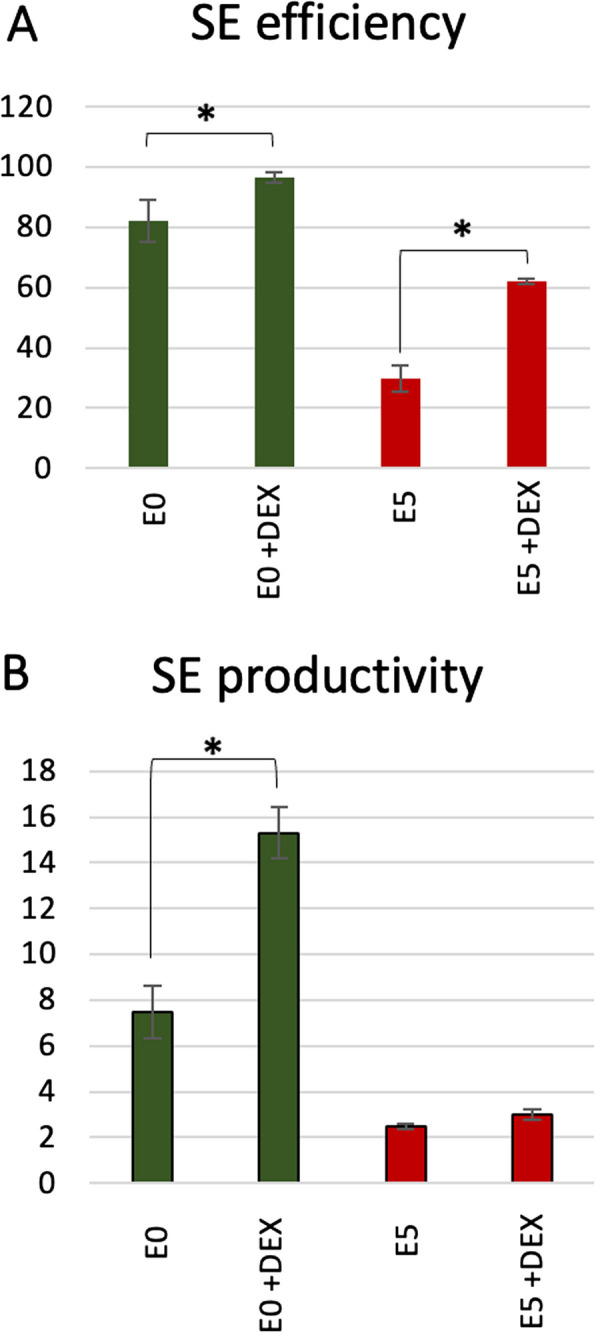
The impact of the polar auxin inhibitor NPA on the embryogenic potential of the *35S::WOX5-GR* line. SE efficiency (**A**) and SE productivity (**B**) were evaluated in explants cultured on auxin-free (E0) and auxin (E5) medium supplemented with NPA. DEX was added to the media to induce *WOX5* overexpression. Asterisk (*)—significantly different values between DEX-treated and DEX-free culture. (*P* < 0.05; *n* = 3 ± standard error)

To further investigate auxin-related functions of *WOX5* in SE in the PIN-dependent auxin transport, we analyzed embryogenic responses of *35S::WOX5-GR* treated with an inhibitor of the polar auxin transport (PAT) in plants, the N-1-naphthylphthalamic acid (NPA) [42]. We found that irrelevantly to *WOX5* expression level, NPA induced SE response in the explants cultured in auxin-free medium (Fig. 7). However, *WOX5* overexpression significantly improved the embryogenic responses of the NPA-treated explants. Interestingly, NPA supplementation of the auxin (E5) medium promoted SE induction in *WOX5-GR* explants, mainly forming non-embryogenic callus on this medium free of NPA (Fig. 1A). The results suggested the role of PAT in SE induction and the involvement of WOX5 in this mechanism.

### Candidate targets of WOX5 involved in the SE induction

#### TF genes regulating biosynthesis and transport (PLT3 and LEC2) and signaling (ARF5) of auxin

**Fig. 8:**
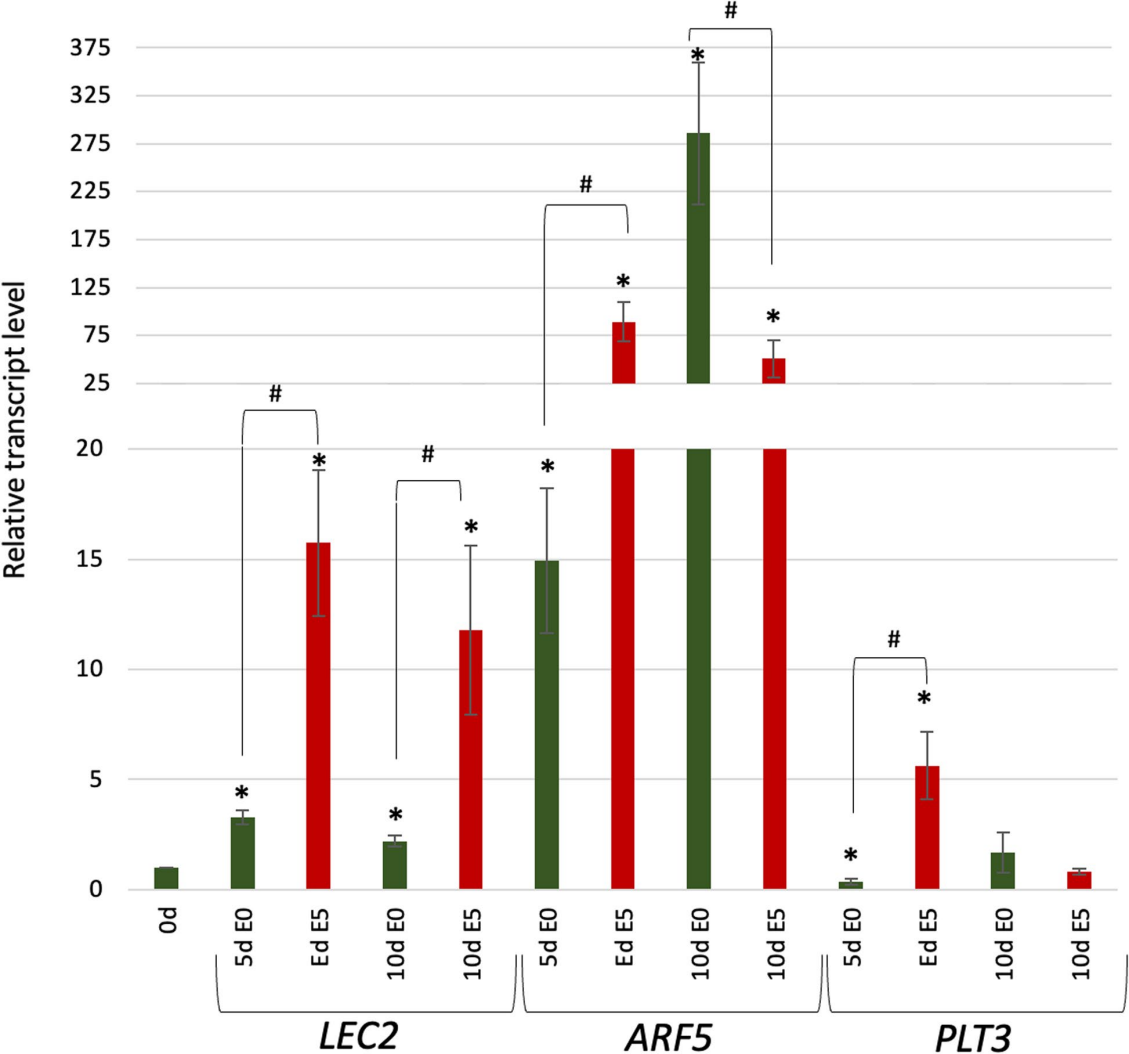
*WOX5* regulates the auxin-related TF genes *LEC2, ARF5,* and *PLT3*. Expression level of *LEC2, ARF5,* and *PLT3* in the *35S::WOX5-GR* explants cultured on auxin-free (E0; green) and auxin (E5; red) media supplemented with DEX. The relative transcript level was normalized to the internal control (*At4g27090*). Statistical analyses were performed using two-way ANOVA (*P* < 0.05) followed by Tukey's honestly significant difference test (Tukey HSD-test) (*P* < 0.05) Asterisks (*)—values significantly different to 0 d (freshly isolated explants – start of culture); hash (#)—significant differences between E5 + DEX and E0 + DEX culture of the same age (*P* < 0.05; *n* = 3 ± standard error); d -day of SE culture

In stem cell maintenance in the RAM, the regulatory relationships of WOX5 with PLETHORA (PLT) TFs and ARF5 were reported [18, 43]. Given that PLTs and ARF5 might control SE induction via regulating LEC2 and auxin biosynthesis [44, 45], we examined the regulatory relationship between WOX5 and the *ARF5, PLT3,* and *LEC2* genes. To this end, expression levels of the candidate targets were analyzed in the explants of the *35S::WOX5-GR* line (Fig. 8). The results showed that the *WOX5* overexpression positively impacted *PLT3*, *LEC2*, and *ARF5* transcript levels in the cultured explants, irrelevantly to auxin presence (E5) of a lack (E0) in the medium. Noteworthy, during the SE induction stage (5 d), the expression levels of all analyzed targets under *WOX5* overexpression were higher on the auxin (E5) compared to the auxin-free medium (E0) (Fig. 8).

**Fig. 9:**
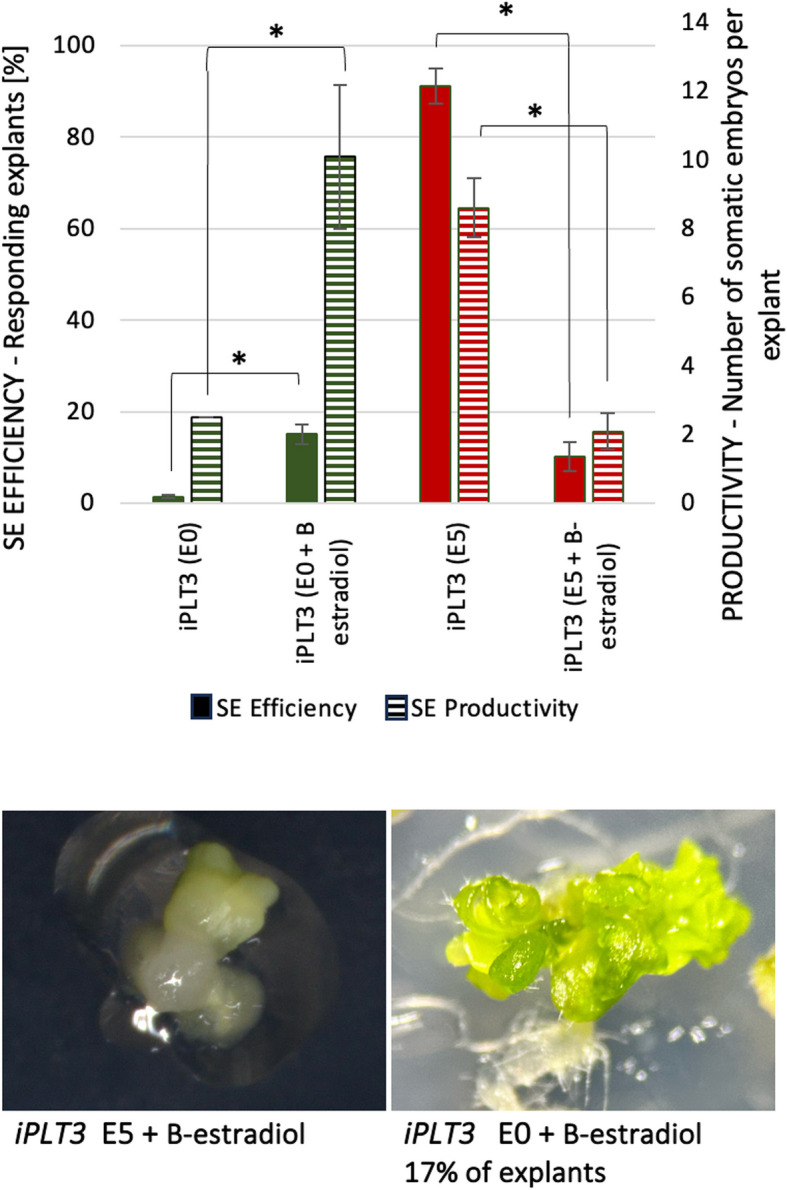
The embryogenic potential of the *PLT3* overexpression line, iPLT3. The iPLT3 explants were cultured on an auxin E5 (red) and auxin-free E0 (green) medium. To induce *PLT3* overexpression, the media were supplemented with β-estradiol. Asterisks (*) – values significantly different in the presence of β-estradiol. β-estradiol (*P* < 0.05; *n* = 3 ± standard error). Solid bars: SE efficiency; Striped bars: SE productivity

Given the assumed auxin-related functions of WOX5 in SE induction, we analyzed the auxin impact on the embryogenic response of the iPLT3 explants overexpressing *PLT3*, a potential WOX5 target. We found that auxin presence in the medium distinctly modulated the embryogenic capacity of the *PLT3* overexpressing culture (Fig. 9). Similarly to *WOX5*, the overexpression of *PLT3* promoted SE induction in auxin-free E0 culture, and the auxin treatment decreased the embryogenic potential of the iPLT3 explants.

The results indicated that the regulatory relationship of *WOX5* and TF genes such as *ARF5*, *LEC2*, and *PLT3* engaged in signaling, biosynthesis, and auxin transport might contribute to the SE induction mechanism.

#### Differentiation factor gene *CDF4* controlling the root stem cell niche

**Fig. 10:**
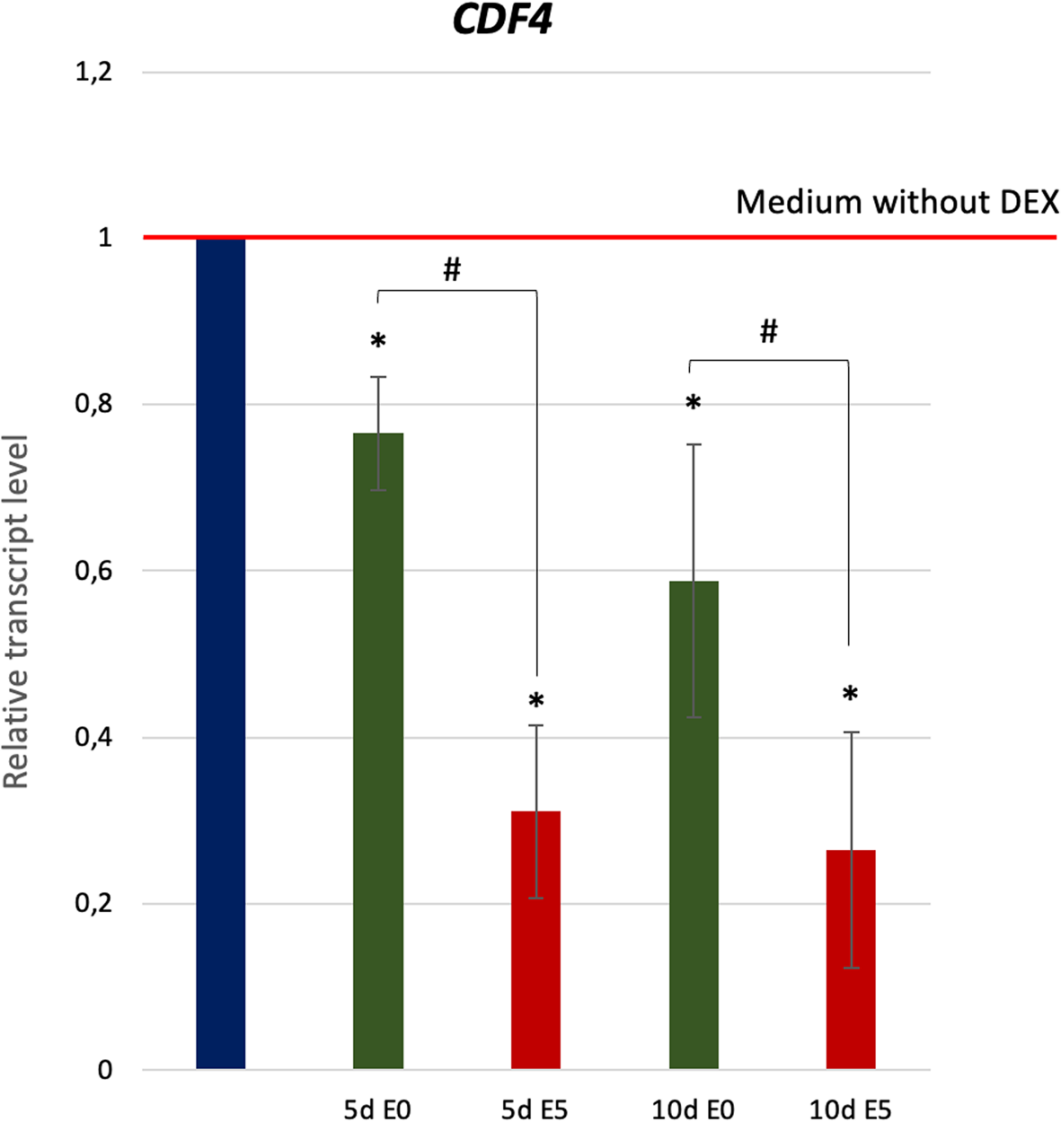
Expression level of *CDF4* in 35S::WOX5-GR explants cultured on auxin-free (E0; green) and auxin (E5; red) media supplemented with DEX. The relative transcript level was normalized to the internal control (*At4g27090*). Statistical analyses were performed using two-way ANOVA (*P* < 0.05) followed by Tukey's honestly significant difference test (Tukey HSD-test) (*P* < 0.05) Asterisks (*)—values significantly different to control medium without DEX; hash (#)—significant differences between E5 + DEX and E0 + DEX culture of the same age (*P* < 0.05; *n* = 3 ± standard error); d—day of SE culture

We also asked if, similarly to the root stem cell niche network [46], WOX5 might control the differentiation factor gene *CDF4* in the embryogenic transition of somatic cells. To verify this assumption, we analyzed the level of *CDF4* transcripts in response to *WOX5* overexpression (Fig. 10). The results showed that *CDF4* transcription is under a negative WOX5 control. DEX-induced overexpression of *WOX5* resulted in a significant decrease of *CDF4* transcripts, and the repressive effect was much higher in the auxin-induced culture (E5). A negative regulatory impact of *WOX5* on *CDF4* also suggested the reverse transcription level of these TFs in the embryogenic culture of WT (Supplementary Fig. 2).

**Fig. 11:**
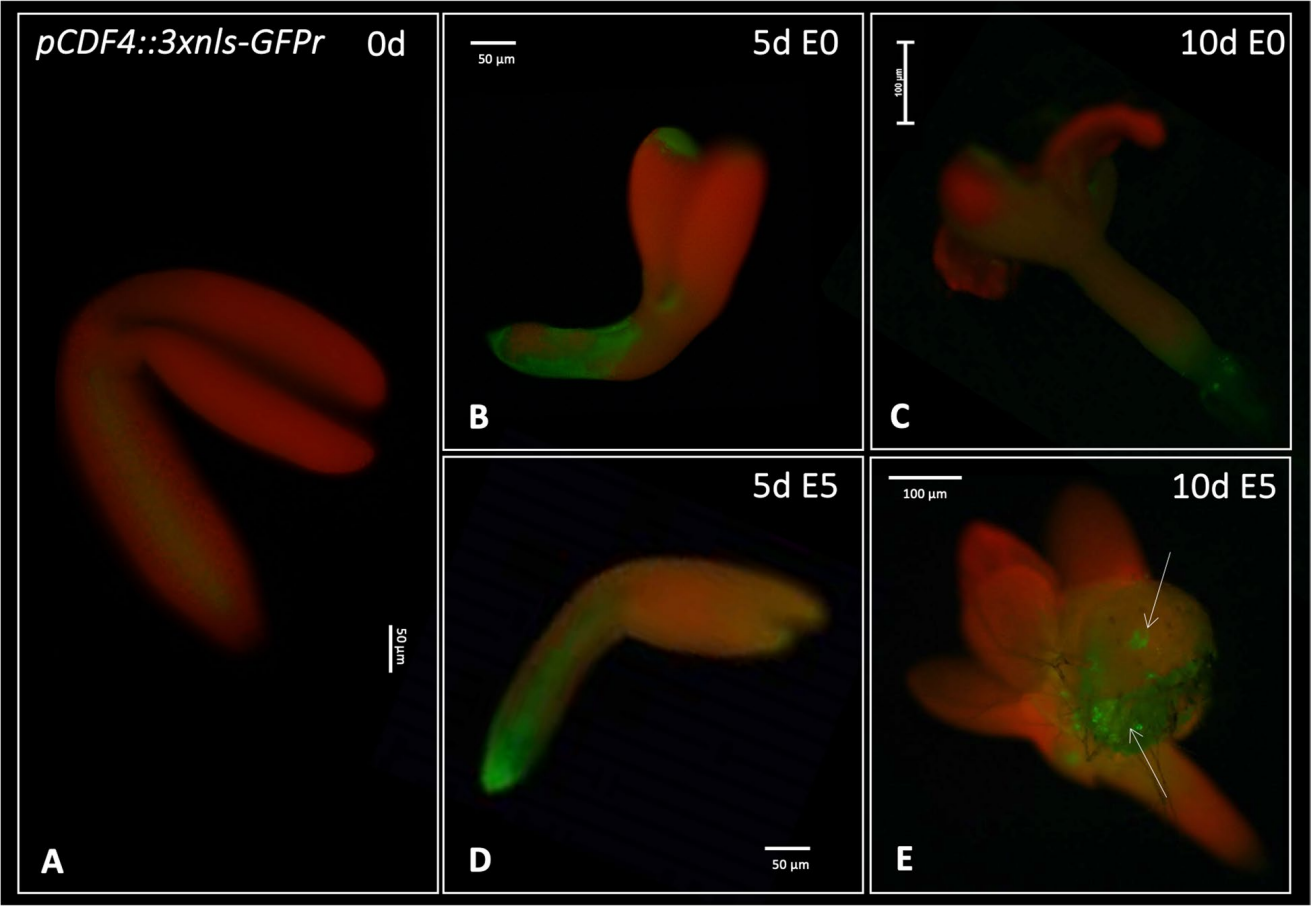
Spatio-temporal expression pattern of *CDF4* in explants cultured on auxin-free E0 (B, C) and auxin E5 (D, E) medium at 0, 5, and 10 d. Arrows indicate the GFP signal associated with the SE-involved tissue; d—day of SE culture. The scale bar indicates 10 μm

We also analyzed the *CDF4* expression pattern in the *pCDF4::3xnls-GFP* explants induced on E0 and E5 media. In freshly isolated explants, GFP signals were not detected (Fig. 11). In contrast, we indicated *CDF4* expression in the root and hypocotyl part of the explants induced for 5 d on both E0 and E5 media. More advanced explant culture (10 d) resulted in the activation of the *CDF4* expression in SE-involved cotyledon tissue, suggesting the engagement of *CDF4* in the advanced stage of embryogenic transition.

**Fig. 12:**
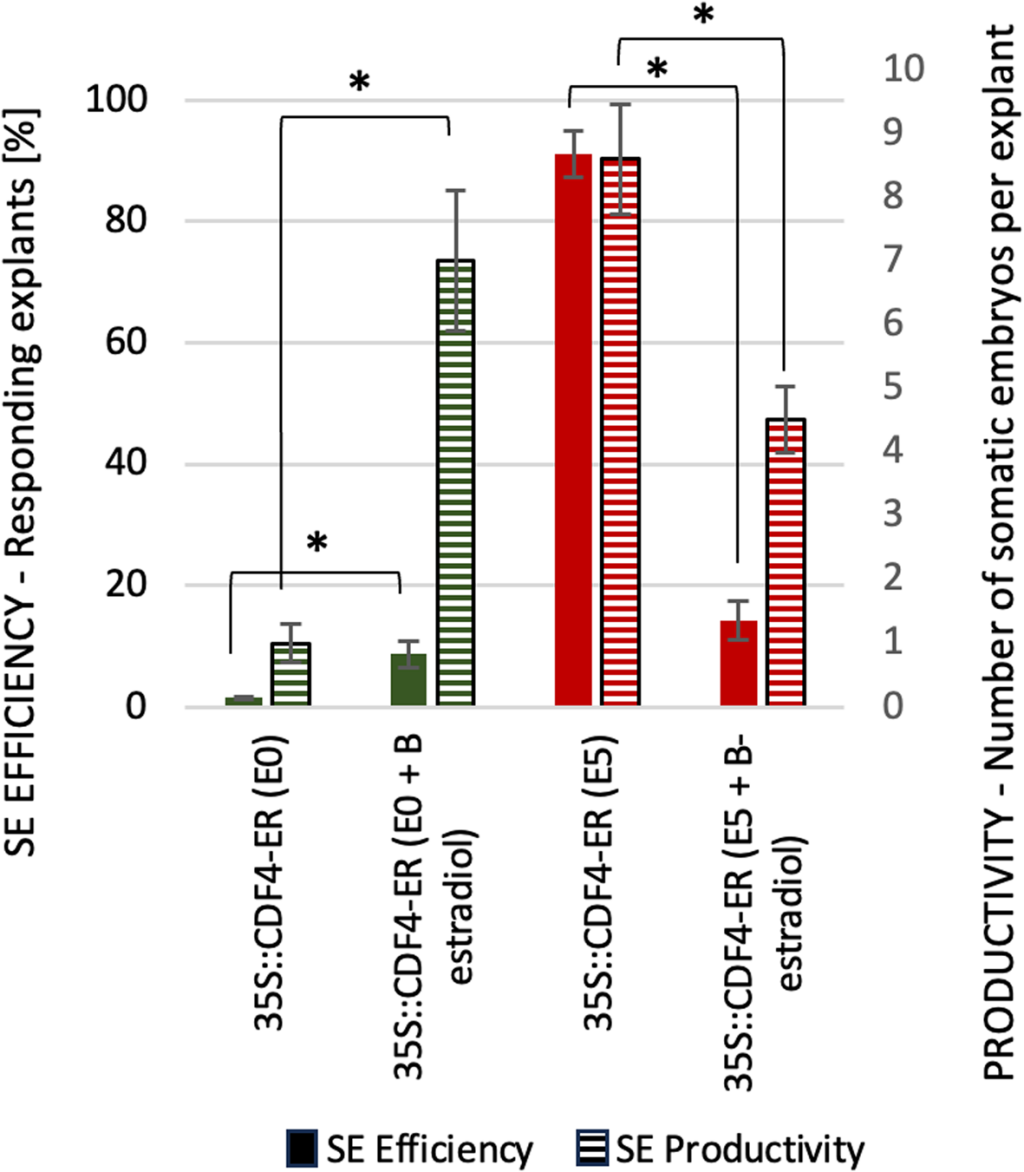
The embryogenic potential of *CDF4* overexpression line, 35S::CDF4-ER. The *CDF4* overexpression was induced with β-estradiol. The transgenic explants were cultured on an E5 (red) and E0 (green) medium with and without the β-estradiol. Asterisks (*) – values significantly different in the presence of β-estradiol (*P* < 0.05; *n* = 3 ± standard error). Solid bars: SE efficiency; Striped bars: SE productivity

Moreover, the role of *CDF4* in SE induction indicated the results on the embryogenic potential of the 35S::CDF4-ER explants. Induced by β-estradiol, the overexpression of *CDF4* significantly inhibited the embryogenic potential of the transgenic explants cultured on auxin E5 medium (Fig. 12). Interestingly, the induction (+ β-estradiol) of *CDF4* overexpression slightly enhanced embryogenic response of the explants in E0 culture.

## Discussion

### WOX5 promotes SE through auxin-related mechanisms

Wild-type (WT) cultures of different plants, including Arabidopsis, usually require auxin treatment, mostly 2,4-D treatment, to induce SE [9, 47]. Our findings that the explants overexpressing *WOX5* can undergo SE induction without 2,4-D treatment pointed to the auxin-related function of WOX5 in SE. Callus instead SE induced by *WOX5* overexpression on auxin media parallels the effects observed in *LEC2* overexpressing culture in which over-optimal auxin accumulation inhibited embryogenic transition and stimulated callus formation [40, 48, 49]. Furthermore, the *WOX5* expression in the cultured explants showed a SE-specific pattern [38] and co-localized with DR5-controlled GFP signals, implying the auxin-related mechanism of WOX5 function in SE.

In support of this, the gene expression results suggest that WOX5 promotes auxin biosynthesis in cultured explants by upregulating *TAA1* and *YUC1,* encoding key enzymes of the TAA1-YUC auxin biosynthesis pathway critical for SE induction [40]. Parallel to SE, WOX5 controls the *TAA1*-mediated auxin biosynthesis in the distal meristem, and QC in root development, where YUC1, acting downstream of TAA1, was suggested as a WOX5 target [35, 50, 51].

Previous studies linked the requirement of auxin gradients to embryogenic transition by indicating the role of polar auxin transport (PAT) and PIN efflux auxin transporters, a central role in plant developmental plasticity and SE [24, 52, 53]. Our findings extend this knowledge by demonstrating that *PIN1* gene expression seems to be activated by *WOX5* overexpression and the inhibition of PAT by NPA, a potent PAT inhibitor [42], might compensate for the need for auxin treatment to induce SE in Arabidopsis explants. The NPA-induced embryogenic response might result from the disturbed PAT, resulting in the re-distribution of auxin in the explants. As a result, auxin accumulates in specific explant cells, mimicking the effect of external 2,4-D application, triggering embryogenic transition [47]. We also revealed the role of WOX5 in SE-related PAT driven by PIN transporters, and we showed that the SE efficiency of NPA-treated explants was enhanced by *WOX5* overexpression. Consistent with PAT-related WOX5 function in SE, WOX5 was reported to affect the localization of PIN proteins in the root tip, contributing to the establishment of auxin maximum in the QC of a critical function for stem cell niche maintenance [35]. Thus, we assumed that WOX5 might create local auxin maxima in the explants by facilitating PIN-dependent auxin redistribution to SE-involved explant parts. Multifaceted regulatory interactions regulate *PIN* gene expression in plant development, and within transcriptional regulators, SE-engaged TFs, WUS, and BBM/PLT were indicated to control *PINs* by a complex network of feedback loops [53]. Within PIN’s regulators, *OsWOX3A* was also implicated in regulating auxin transport genes in the root development of rice [54]. Further analysis is required to verify the regulatory relationships between WOX5 and PINs, focusing on SE-involved PIN1 [24, 47].

### WOX5, a new upstream regulatory element of the TF network in SE

Within the genes interacting with WOX5 in the SE-regulatory network, we assumed *ARF5, LEC2,* and *PLT3* TFs. The central function of ARF5 in auxin signaling controlling almost all developmental processes, including SE induction, has been well documented, but little is known about genetic and epigenetic factors ensuring strict control of *ARF5* expression in different developmental and tissue-specific contexts (reviewed in [55]). The basic mechanism of ARF5 regulation involves an auxin-dependent dynamic feedback loop between ARF5 and Aux/IAA [56]; however, other mechanisms and not yet identified TFs might also control ARF5 activity in response to developmental cues [57]. The results imply that WOX5 is a candidate activator of *ARF5* expression in SE. The direct regulation of *ARF5* by WOX5 cannot be ruled out due to the presence of a WUS-binding box in the *ARF5* promotor [58].

Notably, ARF5 was postulated in SE to control a central SE-regulator, LEC2 TF [40, 44, 59], which we assumed to be under WOX5 control (present results). LEC2 activates auxin biosynthesis by controlling SE's TAA1/YUC pathway [44, 60]. Thus, we assumed that WOX5 might promote auxin biosynthesis in SE by regulating *LEC2*. The present results suggest that WOX5 might also control *PLT3* in SE. Consistent with it, *WOX5* and *PLT3* expression overlapped in root stem cells [43]. Due to the overlapping and compensatory roles of PLT proteins in various aspects of plant development, including SE [45, 61, 62], we assumed that PLT3, similar to its close relative BBM/PLT4, might also regulate *LEC2* to promote auxin biosynthesis and SE induction [63]. A possible scenario for WOX5-mediated regulatory interactions underlying SE induction involves WOX5-stimulated *PLT3* expression followed by activation of LEC2 that stimulates auxin biosynthesis. Future studies should focus on revealing the possibly complex and reciprocal regulatory interactions between WOX5 and other TFs of auxin-related gene networks in SE. The ChiP-seq analysis optimized for low-input procedure as tissue undergoing SE in Arabidopsis might reveal the direct/indirect regulation of *WOX5* targets.

### Repression of *CDF4* by WOX5 in the embryogenic cell transition

Besides gene activation, WOX5 can also repress gene transcription in plant development [64]. Consistent with its dual regulatory function, our results suggest that WOX5 negatively regulates the differentiation factor *CDF4* during SE induction. The repression of *CDF4* in *WOX5* overexpressing explants, particularly in auxin-induced cultures (E5), indicates that WOX5 might mediate the suppression of differentiation cues to maintain embryogenic potential in explant cells. The spatial expression patterns of *CDF4* in SE-induced explants, restricted to the advanced stage (10 d) of explant culture, align with its hypothesized role in cell differentiation likely associated with the somatic embryo development at 10 d culture [37]. Congruently with the differentiation-promoting function in SE, the *CDF4* gene negatively affected the embryogenic transition of explant cells, and its overexpression inhibited the embryogenic response of the explants. These results are consistent with studies demonstrating the critical balance between pluripotency and differentiation signals in SE [16, 65]. The interaction between WOX5 and *CDF4* highlights a regulatory network where WOX5 suppresses differentiation pathways to sustain embryogenic states.

The WOX5-related mechanism of *CDF4* regulation involves histone acetylation to control chromatin accessibility [46, 64]. In roots, to suppress *CDF4* and cell differentiation, WOX5 recruits histone deacetylase HDA19 [46], of the essential role in the embryogenic reprogramming of somatic cell transcriptome [66, 67]. Noteworthy, the WOX5-TPL/TPR-HDA19 complex controls SE-involved TFs in the stem cell niche in the root [46]. Thus, the involvement of HDA19 in WOX5-mediated negative control of *CDF4* in SE might be worth studying. The critical impact of histone acetylation on transcriptome reprogramming in cultured plant explants [66, 68, 69] encourages elaboration of the role of WOX5 in chromatin modifications in SE.

### WOX5 highlights parallels between SE and RAM regulation

The present study provided new evidence on the versatility of developmental processes controlled in vitro by WOX5, TF of root meristem-specific function *in planta*. Parallel to its function in callus development and shoot regeneration in vitro [26–28, 30], we showed a critical role of WOX5 in the embryogenic reprogramming of somatic plant cells. WOX5’s function in SE induction reveals striking similarities between the molecular regulation of embryogenic somatic cells reprogramming in vitro and the root apical meristem (RAM) control [18]. In the RAM, WOX5 maintains the stem cell niche by repressing differentiation-promoting factors such as CDF4 [37]. Similarly, during SE induction, WOX5 suppresses *CDF4* expression to sustain the embryogenic potential of somatic cells, as demonstrated in our experiments. The interaction of WOX5 with SE-involved TFs (LEC2, PLT3, ARF5) regulating auxin-related processes further underscores the shared molecular pathways controlling SE induction and RAM. In both cases, auxin gradients are critical for maintaining the balance between stem cell maintenance and differentiation [24, 70]. Thus, parallel to RAM, WOX5 might integrate hormonal and transcriptional cues and modulate auxin biosynthesis, transport, and signaling to promote pluripotency-like states and embryogenic transition in somatic cells cultured in vitro.

We postulate the significance of WOX5 in controlling auxin-dependent plant developmental plasticity. WOX5 seems to function as a molecular switch that, under specific culture conditions, i.e., explant type and/or hormonal treatment, directs somatic cells to alternative developmental pathways such as callus, shoot regeneration, and SE induction. We postulate *WOX5* as a new candidate for ectopically expressed morphogenic genes, improving the plant regeneration efficiency of in *vitro* culture recalcitrant species [71], as the first attempt was already performed in order to improve wheat genetic transformation using *TaWOX5 * [72]

## Conclusions

**Fig. 13:**
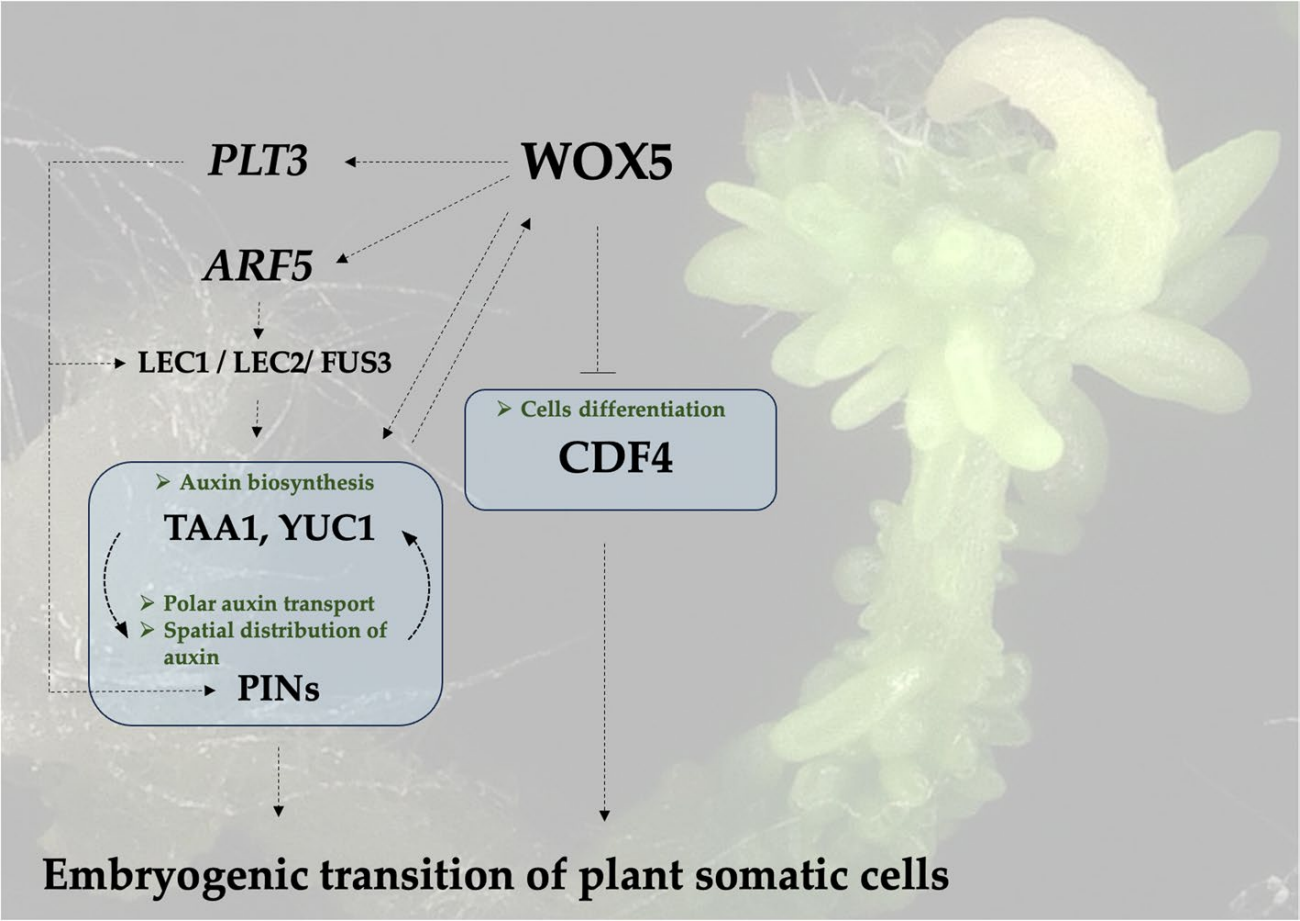
WOX5 emerges as a master regulator of embryogenic potential in somatic cells. Its postulated functions in the transcriptional network governing SE induction integrate auxin biosynthesis, transport, and signaling with the repression of differentiation factors. Auxin stimulates WOX5 [73], while WOX5 activates the auxin biosynthesis by regulating the SE-involved TAA1 gene (the present results; [35, 40]). The WOX5-TAA1 interaction may be acquired by the WOX5 activation of *PLT3* and *ARF5* (present results), which positively regulate the *PINs* [35] and *LEC* genes [44], respectively. The SE-promoted activity of WOX5 also involves the repression of the *CDF4* gene (present results, [46]). WOX5 (WUSCHEL RELATED HOMEOBOX5); PLT3 (PLETHORA3); ARF5 (AUXIN RESPONSE FACTOR5); LEC1, LEC2 (LEAFY COTYLEDON), FUS3 (FUSCA3); TAA1 (TRYPTOPHAN AMINOTRANSFERASE OF ARABIDOPSIS1); PIN (PIN-FORMED); CDF4 (one of DNA‐binding with one finger (DOF) family proteins). Solid line – direct, verified interaction; striped line – indirect, unverified interaction

The study demonstrates WOX5 as a new regulator of auxin-induced SE that controls the transcription of key genes involved in auxin biosynthesis (*TAA1, YUC1*), transport (PIN1), and signaling (*ARF5*) in SE. WOX5 seems to act upstream to other TF regulators of the SE regulatory network, LEC2 and PLT3, to control auxin biosynthesis. Furthermore, by repressing the differentiation factor *CDF4*, WOX5 maintains an undifferentiated, pluripotent state in cells necessary for SE induction. These findings provide new insights into the regulatory SE network and place WOX5 upstream of other major plant regulators of embryo identity and totipotency of somatic cells (Fig. 13). However, additional analysis is needed to verify the direct or indirect relations.

Dissecting the molecular mechanisms underlying WOX5’s regulation of downstream targets, such as LEC2, PLT3, and CDF4, could yield valuable insights into the interplay between auxin, pluripotency, and differentiation signals in plant systems. The similarity of WOX5 functions in SE induction to stem cell maintenance in the root (RAM) highlights the conserved regulatory framework governing pluripotency and differentiation of cells across plant developmental contexts. Future research should explore the potential of WOX5-mediated SE induction to enhance in vitro plant regeneration of essential crops, including cereals.

## Materials and methods

### Plant material and growth conditions

Plants of *Arabidopsis thaliana* (L.) Heynh. Col-0 (WT) and insertional mutant in *WOX5* (*wox5-1, SALK_038262C* – N657590) were studied. Different transgenic plants of Arabidopsis kindly provided by other researchers and institutions were also used. The overexpression lines include DEX-induced *WOX5* (*35S::WOX5-GR*) from Thomas Laux (Institute of Biology III, University of Freiburg, Germany); β-estradiol-induced *CDF4* (*35S::CDF4-ER*) from TRANSPLANTA collection (N2101415), and PLT3 (*iPLT3-mv*) from Yvonne Stahl (Goethe University Frankfurt; [43]). The analyzed reporter lines involved *pWOX5::GFP* from Federico Lopez-Moya (Laboratory of Plant Pathology, Alicante, Spain); *DR5:GFP 35S::WOX5-GR* from Viktoriya V. Lavrekha (Institute of Cytology and Genetics, Novosibirsk, Russia), and *pCDF4:3xnlsGFP* from Thomas Laux (Institute of Biology III, University of Freiburg, Germany).

The seeds were sown in 42-mm-diameter Jiffy-7 peat pots (Jiffy), and plants were grown in a ‘walk-in’ type phytotron under controlled conditions: 22 °C, 16 h/8 h (light/dark) and a light intensity of 100 µE/m^2^s. Cultures grown in vitro were maintained in a controlled growth chamber at 22 °C, 16 h/8 h (light/dark), and a light intensity of 50 µE/m^2^s.

### Somatic embryogenesis induction in vitro

According to standard protocol, Arabidopsis Immature zygotic embryos at the late cotyledonary stage of different *Arabidopsis thaliana* (L.) Heynh genotypes were used as the explants for SE induction under in vitro cultures [74]. Explants were excised from siliques 12–16 days after pollination, sterilized with 20% commercial bleach for 20 min (with sodium hypochlorite), and washed thoroughly with sterile water (3 × 10 min). Sterile explants were cultured on an E5 solid medium containing B5 basal medium (Gamborg et al. 1968) and supplemented with 5.0 µM 2,4-D (2,4-dichlorophenoxyacetic acid, Sigma), 20 g L^−1^ sucrose, and 8 g L^−1^ agar (Oxoid, Hampshire, United Kingdom). In some experiments, a solid medium without auxin—E0 was used. The explant capacity for SE was evaluated after 21 days of culture, and two parameters were evaluated: SE efficiency—the percentage of explants that formed somatic embryos, and SE productivity—the average number of somatic embryos produced by embryogenic explants.

To induce *WOX5* overexpression in the *35S::WOX5-GR* line, explants were transferred to the plates containing medium supplemented with 15 μM DEX. For the control, we used media without DEX. To induce *CDF4* and *PLT3* overexpression in the lines *35S::CDF4-ER* and *iPLT3*, explants were transferred onto a medium supplemented with 10 μM β-estradiol. For the control, we used media without β-estradiol.

The culture combinations were evaluated in three replicates, and at least 30 explants (ten explants/Petri dish) were analyzed per replicate.

### Analysis of gene expression

A *mir*Vana™ Isolation Kit was used to isolate total RNAs from explants. Depending on the age of the culture, 300 (0-day culture) to 100 (10-day culture) explants were collected for RNA isolation. The concentration and purity of RNA was evaluated with a ND-1000 spectrophotometer (Nano-Drop). RNA was treated with RQ1 RNase-free DNase I (Promega) to avoid DNA contamination following the manufacturer's instructions. First-strand cDNA was produced using a RevertAid First Strand cDNA Synthesis Kit (Fermentas). The reverse transcription product was diluted with water at a 1:4 ratio. RT-qPCR was performed in a 10 L − 1 reaction volume using a LightCycler 480 SYBR Green I Master (Roche). The primers relevant to the genes being studied were used in the RT-qPCR analysis (Table 1). The RT-qPCR reactions were performed as described in Wójcik and Gaj (2016). Primary data analysis was performed using LightCycler Software (Roche). Relative expression levels were calculated and normalized to internal control, and the *At4g27090* gene encoded 60S ribosomal protein. The control gene exhibited a constant expression pattern (C_T_ = 18 ± 1) in all analyzed tissue samples. Three biological repetitions of the plant tissues for the Real-Time RT qPCR analysis were used, and two technical replicates of each repetition were carried out. The relative expression level was calculated using 2^–∆∆CT^, where ∆∆C_T_ represents ∆C_T_^reference condition^—∆C_T_^compared condition^.

**Table 1:** Primer sequences that were used in RT-qPCR gene expression analysis

Gene/molecule	Primer sequence
*WOX5*	FP: AACTGCAGAAAGACTTTTATCTACCAAC RP: AACTGCAGTTCAGATGTAAAGTCCTC
*CDF4*	FP: TGGTCGTGTCGTGGTTGGTATG RP: ATAAGCTCGACTTGGCGAACACC
*ARF5*	FP: GCTCGGGTTGGAAGCTTGTATATG RP: TTACGCATCCCACAAACTCTTCC
*PLT3*	FP: GCTGTTGTGGCTGTGGAAACATC RP: TCTTCCCGTCCATCTATGTCGAG
*TAA1*	FP: TTCGTGGTCAATCTGGATCATGG RP: ACCACGTATCGTCACCGTACAC
*YUC1*	FP: ACCTCGTCCGACATAACGCATC RP: TCCCTTGGCAACACATGAACGG
*PIN1*	FP: GGCATGGCTATGTTCAGTCTTGGG RP: ACGGCAGGTCCAACGACAAATC
*LEC2*	[49]
*At4g27090* control	F—AACCGTTTCGTCGCTCTCTT R—ACGGAGGTTCATGGCGTAAG

### GFP signal detection

Explants of the GFP reporter lines cultured on the E5 and E0 media (with or without DEX) for 0, 5, and 10 days were sampled. The GFP signal was analyzed using a Nikon Eclipse Ni-E/Ni-U fluorescent microscope system. GFP fluorescence was excited using a wavelength of 488 nm (halogen lamphouses with a 100–240 VAC Prior Lumen200). The images were recorded with a Nikon Digital Sight DS-Fi2 with a DS-U3 camera.

### Statistical analysis

The statistical analyses were performed using either the Student t-test or a two-way ANOVA (*p* < 0.05), followed by Tukey's honestly significant difference test (Tukey HSD-test) (*p* < 0.05). The figures show the averages from at least three biological replicates with the standard error.

## Supplementary Information

Additional file 1: Supplementary Fig. 1. Embryo-like structures induced by *WOX5* overexpression on auxin-free medium displayed a bipolar somatic embryo-like nature. The 35S::WOX5-GR explants were cultured on auxin-free E0 medium supplemented with DEX. A – an explant developing numerous embryo-like structures in a 21-day-old culture; B – an embryo-like structure separated from the explant; C – the embryo-like structure developing roots and shoots upon transfer onto ½ MS medium.

Additional file 2: Supplementary Fig. 2. Comparison of the relative transcript level of *WOX5* and *CDF4* genes at 0, 5 th, 10 th day in WT culture. Relative transcript level was normalised to the internal control (At4g27090) and calibrated to the 0 day of culture (*n* = 3; ± standard error). Striped bars: *WOX5*; Solid bars: *CDF4*. Statistical analyses were performed using two-way ANOVA (*P* < 0.05) followed by Tukey's honestly significant difference test (Tukey HSD-test) (*P* < 0.05) to assess the differences between the gene expression at 5 and 10 days of the cultures within an analyzed gene and between genes. Significantly different values to day 0 are indicated by hash (#); asterisks (*) values indicate significant differences between *WOX5* and *CDF4* transcript levels at the same stage of culture (*P* < 0.05; *n* = 3 ± standard error); d, day of SE culture.

## Data Availability

The datasets used and/or analysed during the current study are available from the corresponding author on reasonable request.

## References

[1] Crespi M. Plant transcription links environmental cues and phenotypic plasticity. Transcription. 2020;11:97–9. 33252015 10.1080/21541264.2020.1837498 PMC7714417

[2] Nuzzo F, Moine A, Nerva L, Pagliarani C, Perrone I, Boccacci P, et al. Grapevine virome and production of healthy plants by somatic embryogenesis. Microb Biotechnol. 2022;15:1357–73. 35182024 10.1111/1751-7915.14011 PMC9049623

[3] Chen C, Hu Y, Ikeuchi M, Jiao Y, Prasad K, Su YH, et al. Plant regeneration in the new era: from molecular mechanisms to biotechnology applications. Sci China Life Sci. 2024;67:1338–67. 38833085 10.1007/s11427-024-2581-2

[4] Long Y, Yang Y, Pan G, Shen Y. New Insights Into Tissue Culture Plant-Regeneration Mechanisms. Front Plant Sci. 2022;13:926752. 10.3389/fpls.2022.926752 PMC9280033 35845646

[5] Sugimoto K, Temman H, Kadokura S, Matsunaga S. To regenerate or not to regenerate: factors that drive plant regeneration. Curr Opin Plant Biol. 2019;47:138–50. 30703741 10.1016/j.pbi.2018.12.002

[6] Morinaka H, Coleman D, Sugimoto K, Iwase A. Molecular Mechanisms of Plant Regeneration from Differentiated Cells: Approaches from Historical Tissue Culture Systems. Plant Cell Physiol. 2023;64:297–304. 36546730 10.1093/pcp/pcac172 PMC10016324

[7] Elhiti M, Stasolla C. Transduction of Signals during Somatic Embryogenesis. Plants. 2022;11:178. 10.3390/plants11020178 PMC8779037 35050066

[8] Martínez M, Corredoira E. Recent Advances in Plant Somatic Embryogenesis: Where We Stand and Where to Go? Int J Mol Sci. 2024;25:8912. 10.3390/ijms25168912 PMC11354837 39201598

[9] Wójcik AM, Wójcikowska B, Gaj MD. Current perspectives on the auxin-mediated genetic network that controls the induction of somatic embryogenesis in plants. Int J Mol Sci. 2020;21:1333. 10.3390/ijms21041333 PMC7072907 32079138

[10] Pasternak TP, Steinmacher D. Plant Growth Regulation in Cell and Tissue Culture In Vitro. Plants. 2024;13:1–24. 10.3390/plants13020327 PMC10818547 38276784

[11] Gliwicka M, Nowak K, Balazadeh S, Mueller-Roeber B, Gaj MD. Extensive Modulation of the Transcription Factor Transcriptome during Somatic Embryogenesis in Arabidopsis thaliana. PLoS One. 2013;8:e69261. 10.1371/journal.pone.0069261 PMC3714258 23874927

[12] Salaün C, Lepiniec L, Dubreucq B. Genetic and molecular control of somatic embryogenesis. Plants. 2021;10:1467. 10.3390/plants10071467 PMC8309254 34371670

[13] Daniela Cordeiro S, Canhoto J, Correia S. Regulatory non-coding RNAs: Emerging roles during plant cell reprogramming and in vitro regeneration. Front Plant Sci. 2022;13 November:1–10. 10.3389/fpls.2022.1049631 PMC9684189 36438127

[14] Yuan HY, Kagale S, Ferrie AMR. Multifaceted roles of transcription factors during plant embryogenesis. Front Plant Sci. 2023;14 January:1–15. 10.3389/fpls.2023.1322728 PMC10791896 38235196

[15] Wójcikowska B, Wójcik AM, Gaj MD. Epigenetic regulation of auxin-induced somatic embryogenesis in plants. Int J Mol Sci. 2020;21:2307. 10.3390/ijms21072307 PMC7177879 32225116

[16] Fambrini M, Usai G, Pugliesi C. Induction of Somatic Embryogenesis in Plants: Different Players and Focus on WUSCHEL and WUS-RELATED HOMEOBOX (WOX) Transcription Factors. Int J Mol Sci. 2022;23:15950. 10.3390/ijms232415950 PMC9781121 36555594

[17] Graaff E Van Der, Laux T, Rensing SA. The WUS homeobox-containing (WOX) protein family. Genome Biol. 2009;10:248. 10.1186/gb-2009-10-12-248 PMC2812940 20067590

[18] Sarkar AK, Luijten M, Miyashima S, Lenhard M, Hashimoto T, Nakajima K, et al. Conserved factors regulate signalling in Arabidopsis thaliana shoot and root stem cell organizers. Nature. 2007;446:811–4. 17429400 10.1038/nature05703

[19] Rasheed H, Shi L, Winarsih C, Jakada BH, Chai R, Huang H. Plant Growth Regulators: An Overview of WOX Gene Family. Plants. 2024;13:1–16. 10.3390/plants13213108 PMC11548557 39520025

[20] Wang J, Su Y, Kong X, Ding Z, Zhang XS. Initiation and maintenance of plant stem cells in root and shoot apical meristems. aBIOTECH. 2020;1:194–204. 36303567 10.1007/s42994-020-00020-3 PMC9590467

[21] Zhang Y, Jiao Y, Jiao H, Zhao H, Zhu YX. Two-step functional innovation of the stem-cell factors WUS/WOX5 during plant evolution. Mol Biol Evol. 2017;34:640–53. 28053005 10.1093/molbev/msw263 PMC5400392

[22] Wang D, Ma X, Hao Z, Long X, Shi J, Chen J. Overexpression of Liriodenron WOX5 in Arabidopsis Leads to Ectopic Flower Formation and Altered Root Morphology. Int J Mol Sci. 2023;24:906. 10.3390/ijms24020906 PMC9860802 36674428

[23] Zuo J, Niu QW, Frugis G, Chua NH. The WUSCHEL gene promotes vegetative-to-embryonic transition in Arabidopsis. Plant J. 2002;30:349–59. 12000682 10.1046/j.1365-313x.2002.01289.x

[24] Su YH, Zhang XS. Auxin gradients trigger de novo formation of stem cells during somatic embryogenesis. Plant Signal Behav. 2009;4:574–6. 19820347 10.4161/psb.4.7.8730 PMC2710545

[25] Kadri A, March GG De, Cosson V, Ratet P. Embryogenesis in Medicago truncatula Gaertn. 2021. 10.3390/plants10040715 PMC8067838 33917135

[26] Sugimoto K, Jiao Y, Meyerowitz EM. Arabidopsis Regeneration from Multiple Tissues Occurs via a Root Development Pathway. Dev Cell. 2010;18:463–71. 20230752 10.1016/j.devcel.2010.02.004

[27] Zhai N, Pan X, Zeng M, Xu L. Developmental trajectory of pluripotent stem cell establishment in Arabidopsis callus guided by a quiescent center-related gene network. Development (Cambridge). 2023;150:1–10. 10.1242/dev.200879 36762604

[28] Yang Y, Liu C, Yu Y, Ran G, Zhai N, Pi L. WUSCHEL RELATED HOMEOBOX5 and 7 maintain callus development by promoting cell division in Arabidopsis. Plant Sci. 2023;2024(346):112133. 10.1016/j.plantsci.2024.112133 38795752

[29] Rashid SZ, Kyo M. Ectopic expression of WOX5 dramatically alters root-tip morphology in transgenic tobacco. Transgenic Plant J. 2009;3:92–6.

[30] Lee K, Kim JH, Park OS, Jung YJ, Seo PJ. Ectopic expression of WOX5 promotes cytokinin signaling and de novo shoot regeneration. Plant Cell Rep. 2022;41:2415–22. 36219248 10.1007/s00299-022-02932-4

[31] Tvorogova VE, Fedorova YA, Potsenkovskaya EA, Kudriashov AA, Efremova EP, Kvitkovskaya VA, et al. The WUSCHEL-related homeobox transcription factor MtWOX9-1 stimulates somatic embryogenesis in Medicago truncatula. Plant Cell Tissue Organ Cult. 2019;138:517–27.

[32] Yakovleva DV, Efremova EP, Smirnov KV, Simonova VY, Konstantinov ZS, Tvorogova VE, et al. The WOX Genes from the Intermediate Clade: Influence on the Somatic Embryogenesis in Medicago truncatula. Plants. 2024;13:1–16. 10.3390/plants13020223 PMC10819790 38256776

[33] Ogura N, Sasagawa Y, Ito T, Tameshige T, Kawai S, Sano M, et al. WUSCHEL-RELATED HOMEOBOX 13 suppresses de novo shoot regeneration via cell fate control of pluripotent callus. Sci Adv. 2023;9:eadg6983. 10.1126/sciadv.adg6983 PMC10328406 37418524

[34] Cui G, Zhai Y, Li Y, Zheng L, Li Y. The cleavage of WOX5 by the peptidase DA1 connects cytokinin signaling and root stem cell regulation. Curr Biol. 2024;34:5187–96. 10.1016/j.cub.2024.09.052 39437786

[35] Savina MS, Pasternak T, Omelyanchuk NA, Novikova DD, Palme K, Mironova VV, et al. Cell Dynamics in WOX5-Overexpressing Root Tips: The Impact of Local Auxin Biosynthesis. Front Plant Sci. 2020;11 October:1–13. 33193486 10.3389/fpls.2020.560169 PMC7642516

[36] Gaj MD. Direct somatic embryogenesis as a rapid and efficient system for in vitro regeneration of Arabidopsis thaliana. 2001.

[37] Kong X, Lu S, Tian H, Ding Z. WOX5 is Shining in the Root Stem Cell Niche. Trends Plant Sci. 2015;20:601–3. 26440429 10.1016/j.tplants.2015.08.009

[38] Kurczyńska EU, Gaj MD, Ujczak A, Mazur E. Histological analysis of direct somatic embryogenesis in Arabidopsis thaliana (L.) Heynh. Planta. 2007;226:619–28. 17406890 10.1007/s00425-007-0510-6

[39] Stepanova AN, Robertson-Hoyt J, Yun J, Benavente LM, Xie DY, Doležal K, et al. TAA1-Mediated Auxin Biosynthesis Is Essential for Hormone Crosstalk and Plant Development. Cell. 2008;133:177–91. 18394997 10.1016/j.cell.2008.01.047

[40] Wójcikowska B, Jaskóła K, Gąsiorek P, Meus M, Nowak K, Gaj MD. LEAFY COTYLEDON2 (LEC2) promotes embryogenic induction in somatic tissues of Arabidopsis, via YUCCA-mediated auxin biosynthesis. Planta. 2013;238:425–40. 23722561 10.1007/s00425-013-1892-2 PMC3751287

[41] Karami O, Khadem A, Rahimi A, Zagari N, Aigner S, Offringa R. Transient efflux inhibition improves plant regeneration by natural auxins. Plant J. 2024;118:295–303. 38361343 10.1111/tpj.16682

[42] Teale W, Palme K. Naphthylphthalamic acid and the mechanism of polar auxin transport. J Exp Bot. 2018;69:303–12. 28992080 10.1093/jxb/erx323

[43] Burkart RC, Strotmann VI, Kirschner GK, Akinci A, Czempik L, Dolata A, et al. PLETHORA‐WOX5 interaction and subnuclear localization control Arabidopsis root stem cell maintenance. EMBO Rep. 2022;23:e54105. 10.15252/embr.202154105 PMC9171415 35373503

[44] Wójcikowska B, Gaj MD. Expression profiling of AUXIN RESPONSE FACTOR genes during somatic embryogenesis induction in Arabidopsis. Plant Cell Rep. 2017;36:843–58. 28255787 10.1007/s00299-017-2114-3 PMC5486788

[45] Horstman A, Bemer M, Boutilier K. A transcriptional view on somatic embryogenesis. Regeneration. 2017;4:201–16. 29299323 10.1002/reg2.91 PMC5743784

[46] Pi L, Aichinger E, van der Graaff E, Llavata-Peris CI, Weijers D, Hennig L, et al. Organizer-Derived WOX5 Signal Maintains Root Columella Stem Cells through Chromatin-Mediated Repression of CDF4 Expression. Dev Cell. 2015;33:576–88. 26028217 10.1016/j.devcel.2015.04.024

[47] Karami O, Philipsen C, Rahimi A, Nurillah AR, Boutilier K, Offringa R. Endogenous auxin maintains embryonic cell identity and promotes somatic embryo development in Arabidopsis. Plant J. 2023;113:7–22. 36345646 10.1111/tpj.16024 PMC10098609

[48] Wójcikowska B, Gaj MD. LEAFY COTYLEDON2-mediated control of the endogenous hormone content: implications for the induction of somatic embryogenesis in Arabidopsis. Plant Cell Tissue Organ Cult. 2015;121:255–8.

[49] Kraut M, Wójcikowska B, Ledwoń A, Gaj MD. Immature zygotic embryo cultures of Arabidopsis. Amodel system for molecular studies on morphogenic pathways induced in vitro. Acta Biol Crac Ser Bot. 2011;53:59–67.

[50] Tian H, Wabnik K, Niu T, Li H, Yu Q, Pollmann S, et al. WOX5-IAA17 feedback circuit-mediated cellular auxin response is crucial for the patterning of root stem cell niches in arabidopsis. Mol Plant. 2014;7:277–89. 23939433 10.1093/mp/sst118

[51] Sharma M, Friedrich T, Oluoch P, Peruzzo F, Jha V, Pi L, et al. A coherent feed-forward-loop in the Arabidopsis root stem cell organizer regulates auxin biosynthesis and columella stem cell maintenance. Nat Plants. 2024;10:1737–48. 10.1038/s41477-024-01810-z 39394505

[52] Soriano M, Li H, Jacquard C, Angenent GC, Krochko J, Offringa R, et al. Plasticity in cell division patterns and auxin transport dependency during in vitro embryogenesis in Brassica napus. Plant Cell. 2014;26:2568–81. 24951481 10.1105/tpc.114.126300 PMC4114952

[53] Habets MEJ, Offringa R. PIN-driven polar auxin transport in plant developmental plasticity: A key target for environmental and endogenous signals. New Phytol. 2014;203:362–77. 24863651 10.1111/nph.12831

[54] Yoo S-C, Cho S-H, Paek N-C. Rice WUSCHEL-related homeobox 3A (OsWOX3A) modulates auxin-transport gene expression in lateral root and root hair development. Plant Signal Behav. 2013;8:e25929. 24002214 10.4161/psb.25929 PMC4091085

[55] Wójcikowska B, Belaidi S, Robert HS. Game of thrones among AUXIN RESPONSE FACTORs - over 30 years of MONOPTEROS research. J Exp Bot. 2023;74:6904–21. 37450945 10.1093/jxb/erad272 PMC10690734

[56] Krogan NT, Yin X, Ckurshumova W, Berleth T. Distinct subclades of Aux/IAA genes are direct targets of ARF5/MP transcriptional regulation. New Phytol. 2014;204:474–83. 25145395 10.1111/nph.12994

[57] Cavalleri A, Astori C, Truskina J, Cucinotta M, Farcot E, Chrysanthou E, et al. Auxin-dependent post-translational regulation of MONOPTEROS in the Arabidopsis root. Cell Rep. 2024;43:115083. 39675001 10.1016/j.celrep.2024.115083

[58] Ma Y, Miotk A, Šutiković Z, Ermakova O, Wenzl C, Medzihradszky A, et al. WUSCHEL acts as an auxin response rheostat to maintain apical stem cells in Arabidopsis. Nat Commun. 2019;10:1–11. 31704928 10.1038/s41467-019-13074-9 PMC6841675

[59] Stone SL, Braybrook SA, Paula SL, Kwong LW, Meuser J, Pelletier J, et al. Arabidopsis LEAFY COTYLEDON2 induces maturation traits and auxin activity: Implications for somatic embryogenesis. Proc Natl Acad Sci U S A. 2008;105:3151–6. 18287041 10.1073/pnas.0712364105 PMC2268600

[60] Quintana-Escobar AO, Nic-Can GI, Galaz Avalos RM, Loyola-Vargas VM, Gongora-Castillo E. Transcriptome analysis of the induction of somatic embryogenesis in Coffea canephora and the participation of ARF and Aux/IAA genes. PeerJ. 2019;16:7:e7752. 10.7717/peerj.7752 PMC6800528 31637116

[61] Horstman A, Willemsen V, Boutilier K, Heidstra R. AINTEGUMENTA-LIKE proteins: Hubs in a plethora of networks. Trends Plant Sci. 2014;19:146–57. 24280109 10.1016/j.tplants.2013.10.010

[62] Iohannes SD, Jackson D. Tackling redundancy: genetic mechanisms underlying paralog compensation in plants. New Phytol. 2023;240:1381–9. 37724752 10.1111/nph.19267

[63] Horstman A, Li M, Heidmann I, Weemen M, Chen B, Muino JM, et al. The BABY BOOM transcription factor activates the LEC1-ABI3-FUS3-LEC2 network to induce somatic embryogenesis. Plant Physiol. 2017;175:848–57. 28830937 10.1104/pp.17.00232 PMC5619889

[64] Zhang N, Bitterli P, Oluoch P, Hermann M, Aichinger E, Groot E, et al. Deciphering the molecular logic of WOX5 function in the root stem cell organizer. Plant Cell Rep. 2024. 10.1038/s44318-024-00302-2. 39558109 10.1038/s44318-024-00302-2 PMC11696986

[65] Magnani E, Jiménez-Gómez JM, Soubigou-Taconnat L, Lepiniec L, Fiume E. Profiling the onset of somatic embryogenesis in Arabidopsis. BMC Genomics. 2017;18:1–12. 29284399 10.1186/s12864-017-4391-1 PMC5747089

[66] Morończyk J, Braszewska A, Wójcikowska B, Chwiałkowska K, Nowak K, Wójcik AM, et al. Insights into the Histone Acetylation-Mediated Regulation of the Transcription Factor Genes That Control the Embryogenic Transition in the Somatic Cells of Arabidopsis. Cells. 2022;11:863. 10.3390/cells11050863 PMC8909028 35269485

[67] Wójcikowska B, Chwiałkowska K, Nowak K, Citerne S, Morończyk J, Wójcik AM, et al. Transcriptomic profiling reveals histone acetylation-regulated genes involved in somatic embryogenesis in Arabidopsis thaliana. BMC Genomics. 2024;25:788. 10.1186/s12864-024-10623-5 PMC11325840 39148037

[68] Ikeuchi M, Iwase A, Sugimoto K. Control of plant cell differentiation by histone modification and DNA methylation. Curr Opin Plant Biol. 2015;28:60–7. 26454697 10.1016/j.pbi.2015.09.004

[69] Nowak K, Morończyk J, Wójcik A, Gaj MD. AGL15 controls the embryogenic reprogramming of somatic cells in arabidopsis through the histone acetylation-mediated repression of the mirna biogenesis genes. Int J Mol Sci. 2020;21:1–20. 10.3390/ijms21186733 PMC7554740 32937992

[70] Ding Z, Friml J. Auxin regulates distal stem cell differentiation in Arabidopsis roots. Proc Natl Acad Sci U S A. 2010;107:12046–51. 20543136 10.1073/pnas.1000672107 PMC2900669

[71] Gordon-Kamm B, Sardesai N, Arling M, Lowe K, Hoerster G, Betts S, et al. Using morphogenic genes to improve recovery and regeneration of transgenic plants. Plants. 2019;8:38. 10.3390/plants8020038 PMC6409764 30754699

[72] Wang K, Shi L, Liang X, Zhao P, Wang W, Liu J, et al. The gene TaWOX5 overcomes genotype dependency in wheat genetic transformation. Nat Plants. 2022;8:110–7. 35027699 10.1038/s41477-021-01085-8

[73] Sato A, Soeno K, Kikuchi R, Narukawa-Nara M, Yamazaki C, Kakei Y, et al. Indole-3-pyruvic acid regulates TAA1 activity, which plays a key role in coordinating the two steps of auxin biosynthesis. Proc Natl Acad Sci U S A. 2022;119:1–7. 10.1073/pnas.2203633119 PMC9231625 35696560

[74] Gaj MD. Direct somatic embryogenesis as a rapid and efficient system for in vitro regeneration of Arabidopsis thaliana. Plant Cell Tissue Organ Cult. 2001;64:39–46.

