# Supplementary figures and images for: From root to embryogenic transition: WOX5 reprograms plant somatic cells via auxin-mediated pathways
Source: BMC Plant Biol. 2025 May 15;25:642. doi: 10.1186/s12870-025-06687-4 (PMC12080028; doi:10.1186/s12870-025-06687-4)

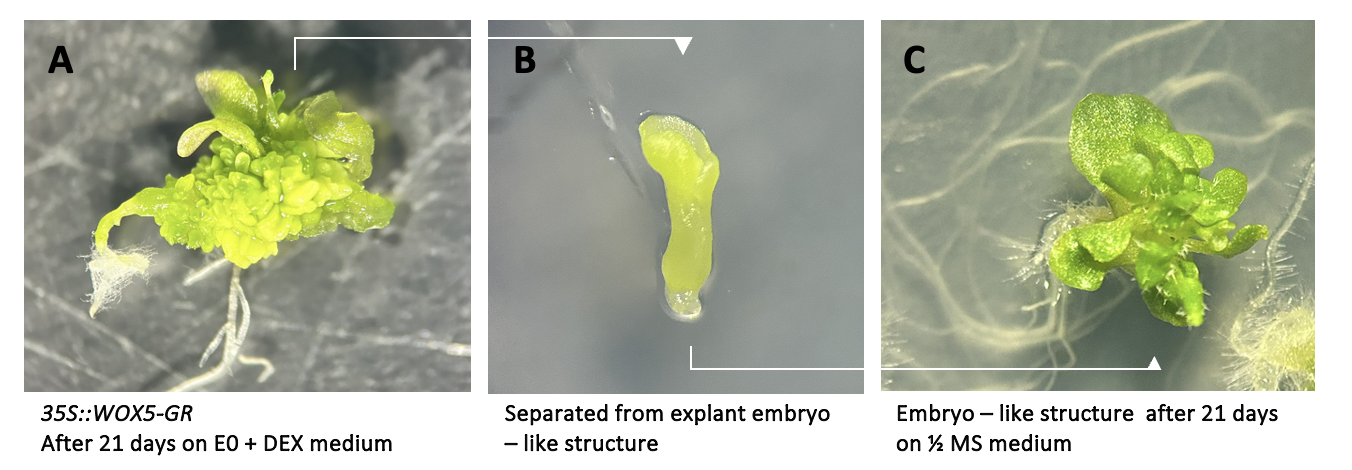

Supplement: Supplementary file 1 — Additional file 1: Supplementary Fig. 1. Embryo-like structures induced by WOX5 overexpression on auxin-free medium displayed a bipolar somatic embryo-like nature. The 35S::WOX5-GR explants were cultured on auxin-free E0 medium supplemented with DEX. A – an explant developing numerous embryo-like structures in a 21-day-old culture; B – an embryo-like structure separated from the explant; C – the embryo-like structure developing roots and shoots upon transfer onto ½ MS medium. [file 12870_2025_6687_MOESM1_ESM.png]

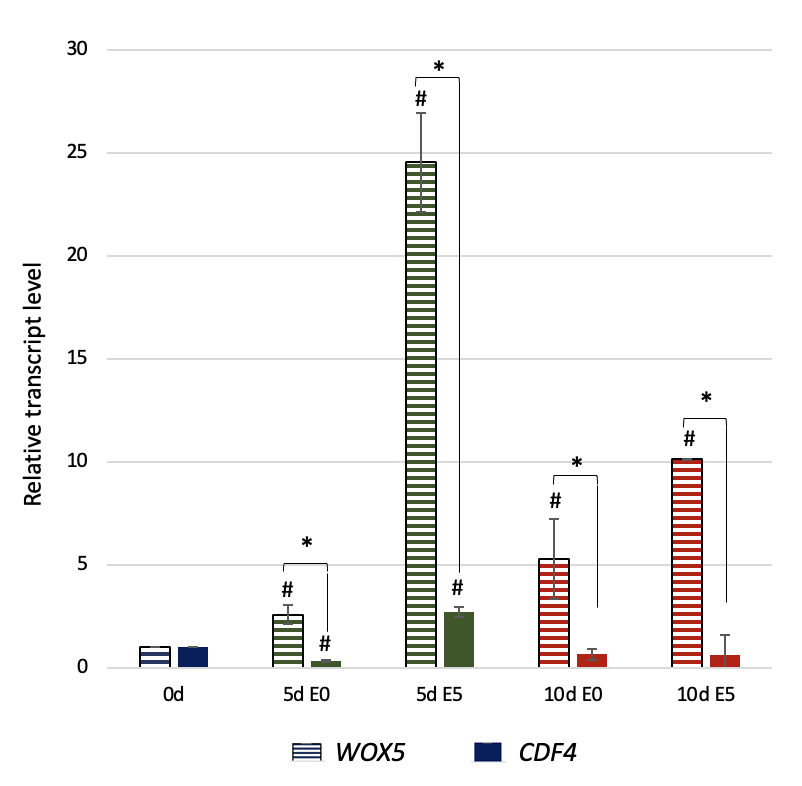

Supplement: Supplementary file 2 — Additional file 2: Supplementary Fig. 2. Comparison of the relative transcript level of WOX5 and CDF4 genes at 0, 5 th, 10 th day in WT culture. Relative transcript level was normalised to the internal control (At4g27090) and calibrated to the 0 day of culture (n = 3; ± standard error). Striped bars: WOX5; Solid bars: CDF4. Statistical analyses were performed using two-way ANOVA (P < 0.05) followed by Tukey's honestly significant difference test (Tukey HSD-test) (P < 0.05) to assess the differences between the gene expression at 5 and 10 days of the cultures within an analyzed gene and between genes. Significantly different values to day 0 are indicated by hash (#); asterisks (*) values indicate significant differences between WOX5 and CDF4 transcript levels at the same stage of culture (P < 0.05; n = 3 ± standard error); d, day of SE culture. [file 12870_2025_6687_MOESM2_ESM.png]
